# Inflammatory markers in postoperative delirium (POD) and cognitive dysfunction (POCD): A meta-analysis of observational studies

**DOI:** 10.1371/journal.pone.0195659

**Published:** 2018-04-11

**Authors:** Xuling Liu, Yang Yu, Shengmei Zhu

**Affiliations:** 1 Zhejiang University School of Medicine, Hangzhou, Zhejiang, P.R. China; 2 Department of Anesthesiology, The First Affiliated Hospital, School of Medicine, Zhejiang University, Hangzhou, Zhejiang, P.R. China; Imperial College London, UNITED KINGDOM

## Abstract

**Background:**

The aim of this study was to summarize and discuss the similarities and differences in inflammatory biomarkers in postoperative delirium (POD) and cognitive dysfunction (POCD).

**Methods:**

A systematic retrieval of literature up to June 2017 in PubMed, Embase, the Cochrane Library, the China National Knowledge Infrastructure database, and the Wanfang database was conducted. Extracted data were analyzed with STATA (version 14). The standardized mean difference (SMD) and the 95% confidence interval (95% CI) of each indicator were calculated using a random effect model. We also performed tests of heterogeneity, sensitivity analysis, assessments of bias, and meta-regression in this meta-analysis.

**Results:**

A total of 54 observational studies were included. By meta-analysis we found significantly increased C-reactive protein (CRP) (9 studies, SMD 0.883, 95% CI 0.130 to 1.637, *P* = 0.022 in POD; 10 studies, SMD -0.133, 95% CI -0.512 to 0.246, *P* = 0.429 in POCD) and interleukin (IL)-6 (7 studies, SMD 0.386, 95% CI 0.054 to 0.717, *P* = 0.022 in POD; 16 studies, SMD 0.089, 95% CI -0.133 to 0.311, *P* = 0.433 in POCD) concentrations in both POD and POCD patients. We also found that the SMDs of CRP and IL-6 from POCD patients were positively correlated with surgery type in the meta-regression (CRP: Coefficient = 1.555365, *P* = 0.001, 10 studies; IL-6: Coefficient = -0.6455521, *P* = 0.086, 16 studies).

**Conclusion:**

Available evidence from medium-to-high quality observational studies suggests that POD and POCD are indeed correlated with the concentration of peripheral and cerebrospinal fluid (CSF) inflammatory markers. Some of these markers, such as CRP and IL-6, play roles in both POD and POCD, while others are specific to either one of them.

## Introduction

An emerging body of evidence suggests that both anesthesia and surgery have long-term effects on cognition. These cognitive impairments have been detected by various neuropsychological tests at different time points after operation. This has given rise to the terms “postoperative cognitive dysfunction” (POCD) and “postoperative delirium” (POD). We refer to both simultaneously as "postoperative cerebral dysfunction".

Delirium is a neuropsychiatric behavioral syndrome, manifested as an altered level of consciousness, inattention, and sleep-wake cycle disturbance. The two main features of delirium are acute onset and fluctuating course [1–3]. POD is age-related, affecting 15%-53% of elderly patients after surgery [4–6].

POCD is a common complication of the central nervous system after surgery, which occurs in patients over 65 years old [7], and mainly manifests as damage to memory, mental capacity, language ability, or other aspects of cerebral function [8]. The World Health Organization has not yet classified this clinical phenomenon as an independent disease, making it difficult to accurately define it [9]. POCD can last from several days to several years.

POD and POCD have been associated with various adverse outcomes, including prolonged hospitalization, increased complications and mortality, and decreased quality of life [10–12]. There is already a great deal of knowledge about the epidemiology of POD and POCD, but they are only clinically diagnosed, without any precise biomarkers to guide diagnosis. However, there is growing evidence that an inflammatory response may play a role in the presence of POD/POCD. Therefore, this meta-analysis aimed to identify the current published literature examining blood or CSF in POD/POCD to summarize the existing knowledge and to determine the relationship between POD/POCD and specific inflammatory markers.

## Materials and methods

### Literature search

Two reviewers searched studies published before 15 June 2017 that examined inflammatory markers in the serum or CSF of patients with POD/POCD. Searches were performed using a comprehensive text-word and Medical Subject Headings-based electronic search of PubMed, Embase, Web of Science, Cochrane, the China National Knowledge Infrastructure database, and the Wanfang database. We used terms including “delirium” or “cognitive dysfunction,” and “inflammation mediators” or “cytokine” (S1 Appendix). Studies were restricted to English and Chinese language publications.

### Study selection

The inclusion criteria were as follows:

1. Study design: case-control or cohort study with non-POD/POCD subjects as controls;
2. POD assessment tool: Diagnostic and Statistical Manual of Mental Disorders (DSM Third Edition; or DSM Fourth Edition) [13], Confusion Assessment Method (CAM) [14], Confusion Assessment Method for the ICU (CAM-ICU), Delirium Rating Scale (DRS) [15], or International Classification of Diseases, Tenth Revision (ICD-10) criteria [16]; POCD assessment tool: Mini-mental State Examination (MMSE) [17] or a series of neuropsychological tests (see “Definitions of POD and POCD” below);
3. Subjects: human;
4. Full text available (detailed information);
5. Data on inflammatory biomarker findings in serum or CSF in POD/POCD patients could be extracted.

The exclusion criteria were:

1. Case reports and reviews;
2. Randomized controlled trials (RCTs) measuring the effects of drugs;
3. No identifiable POD/POCD subgroups;
4. Unavailable data format.

### Data extraction and synthesis

Two reviewers independently screened the search results according to the inclusion criteria and extracted relevant data, which was checked by another reviewer. The following information was tabulated from each paper: (1) author and year of publication; (2) country; (3) study design; (4) sample size and participant characteristics; (5) type of anesthesia; (6) type of surgery; (7) biomarker(s) studied; and (8) method used to diagnose POD/POCD. In cases in which more than one publication of the same trial existed, only the latest publication with the most complete data was included. Disagreements were settled by consensus.

For studies expressing effect sizes as the median with the range or interquartile range, we applied appropriate formulas to converted them to mean and standard deviation (SD). The median can be used to estimate mean when the sample size is larger than 25. When the sample size was moderate (15 < *n* ≤ 70, it was most appropriate to use the formula range/4 [18] to estimate SD. For a larger sample size (*n* > 70), the formula range/6 gives the optimal estimator for the SD. The formula interquartile range/1.35 [19] was used to calculate SD from interquartile ranges.

### Assessment of study quality

Study quality was assessed independently by two of the authors using the Newcastle-Ottawa scale (NOS) [20]. The NOS score ranges from 0–9 stars. A quality score was calculated based on three major components: (1) the selection of study groups (0–4 stars), (2) the comparability of study groups (0–2 stars), and (3) ascertainment of the exposure and outcome of interest in the case-control and cohort studies (0 to 3 stars). Studies with scores of 7–9, 4–6, and 0–3 stars were respectively considered to be of high, medium, and low quality. Disagreement was resolved by discussion and consensus.

### Statistical analyses

Where factors of interest were reported by two or more studies, the standardized mean difference (SMD) and 95% confidence interval (95% CI) were calculated under random effects models (REM) or fixed-effect models (FEM). A Q statistic was calculated using a chi-squared test to quantify the heterogeneity among combined trials, with *P* ≤ 0.10 used for statistical existent heterogeneity. Inconsistency was calculated using an I^2^ index to determine the impact of heterogeneity. In case of the presence of statistical heterogeneity, the REM was used for the analysis. In the absence of statistically significant heterogeneity, the FEM was used.

Subgroup analyses were carried out in order to explore possible sources of heterogeneity. These included the type of surgery and ethnicity. For each variable, subgroup analyses were pre-specified and then meta-analyzed. The overall effect sizes of each member of a subgroup pair were subjected to two-tailed z tests to examine the significance of the difference. Sensitivity analyses were performed to investigate the influence of each individual study on the overall meta-analysis summary estimate and the validity of the effect size. The risk of publication bias was assessed using Egger’s tests. Meta-regression analysis, with *P* ≤ 0.10 indicating statistical significance, was used to test whether effect sizes were influenced by specific study design features.

All data was sorted through Excel 2016, and meta-analyses were undertaken in STATA 14.1 (Stata Corporation, College Station, Texas, USA)

## Results

### Results of literature search

**Fig 1:**
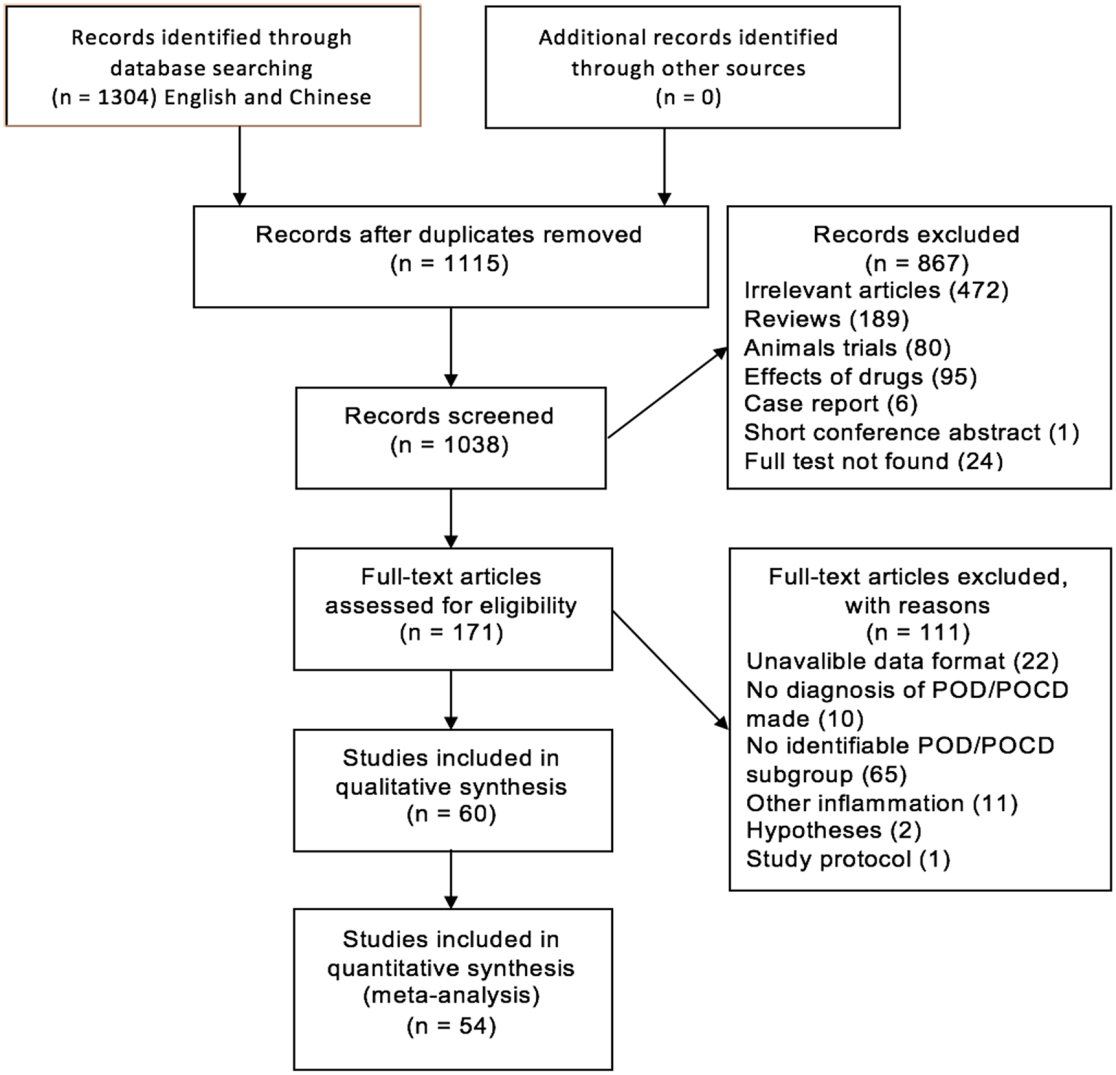
Flow diagram of article selection.

Details of the flow of study identification are shown in Fig 1. Multiple database literature searches led to the identification of 1304 article abstracts. After checking for duplicates, 1115 publications remained, of which 867 were excluded at the screening stage. Of these, 111 publications were excluded at the study eligibility determination stage. The main reasons for the exclusions included: unavailable data format (n = 22); no diagnosis of POD/POCD made (n = 10); no identifiable POD/POCD subgroup (n = 65); other inflammation (11); relevant hypotheses (n = 2); study protocol (n = 1). Thus, 54 studies were included in the meta-analysis, of which 30 were about POD [21–50], 23 about POCD [51–73], and one included both POD and POCD [74]. The major characteristics of the included studies are presented in S1 Table.

The quality of the included studies, in general, ranged from medium to high, when weighed with the NOS (S2 Table). Among the included studies, 10 were case-control studies, and 44 were cohort studies.

### Definitions of POD and POCD

#### Definition of POD

The Confusion Assessment Method (CAM: sensitivity of 94%, specificity of 89% with high inter-rater reliability [75]) is a widely used, standardized method for the identification of delirium. Twenty-five studies applied CAM to define POD [22–27, 29–31, 33–36, 38–40, 42–50] of which three were assessed with CAM and confirmed with the Diagnostic and Statistical Manual of Mental Disorders (Fourth Edition, Text Revision; DSM-IV-TR) [25–27], and one was evaluated through CAM and the Delirium Rating Scale [24] (DRS: sensitivity of 82% and specificity of 94%, cut-off score of 10 [15]). Two studies used DRS to define delirium [21, 28]. In one study, delirium was diagnosed using DSM-IV and DRS [32].

#### Definition of POCD

Unlike for POD, clear diagnostic criteria are lacking for POCD [76]. Changes in cognitive function were measured before and after surgery using standardized neuropsychological assessment tools. Twelve studies assessed the neuropsychological state of patients using a battery of neuropsychological tests [51, 52, 54–57, 60, 62, 65, 68, 69, 73] (see S1 Table for details). In these studies, “the 1 SD-criterion” (a decline of more than 1 SD from baseline) and “a combined Z-score” (at least two Z-scores for single test parameters >2 or the combined Z-score >2) were used as diagnostic criteria for POCD [76]. The other twelve studies used the raw change on the MMSE [53, 58, 59, 61, 63, 64, 66, 67, 69, 71, 72, 74], a fast cognitive screening instrument [17], to determine the incidence of POCD.

### Cytokine concentrations

**Fig 2:**
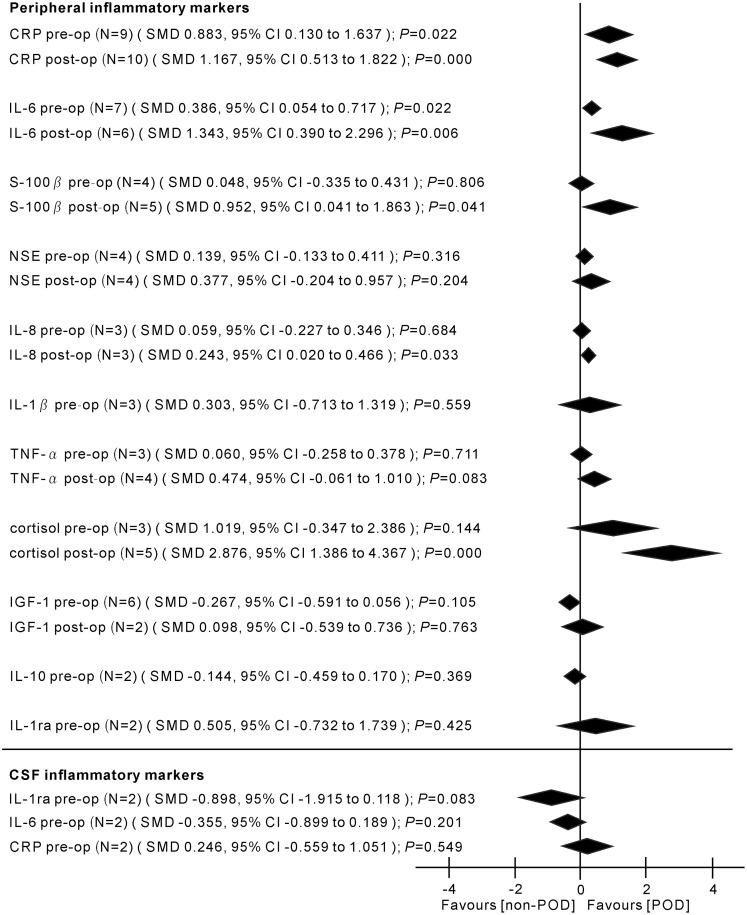
Results of the random-effects meta-analysis for the association between inflammatory markers and POD. Positive values indicate higher levels in POD patients; negative values indicate higher levels in control patients. CRP, C-reactive protein; IL-6, interleukin-6; S-100β, S-100 calcium binding protein beta subunit; NSE, neuron-specific enolase; IL-8, interleukin-8; IL-1β, interleukin-1 beta; TNF-α, tumor necrosis factor alpha; IGF-1, insulin-like growth factor-1; IL-10, interleukin-10; IL-1ra, interleukin-1 receptor antagonist; pre-op, pre-operation; post-op, post-operation; N, number of studies; SMD, standardized mean difference; CI, confidence interval.

**Fig 3:**
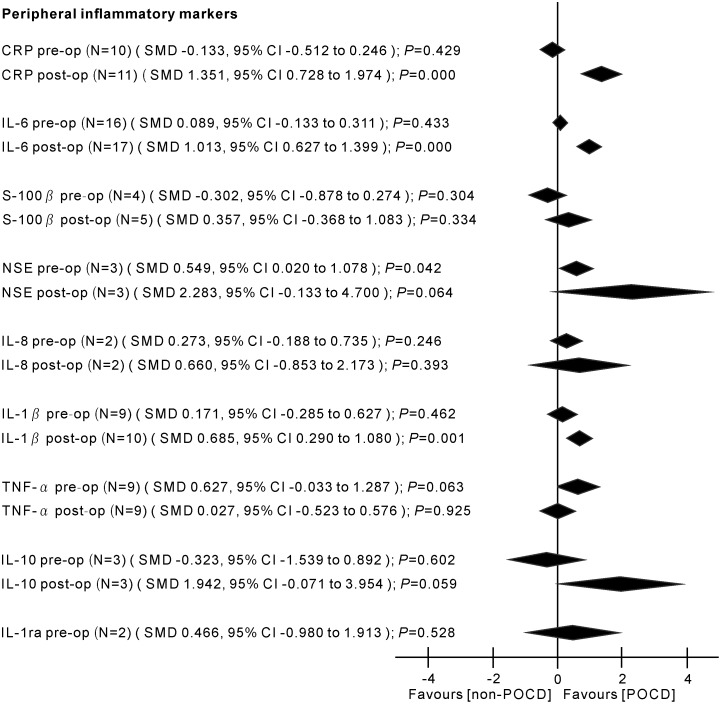
Results of the random-effects meta-analysis for the association between inflammatory markers and POCD. Positive values indicate higher levels in POCD patients; negative values indicate higher levels in control patients. CRP, C-reactive protein; IL-6, interleukin-6; S-100β, S-100 calcium binding protein beta subunit; NSE, neuron-specific enolase; IL-8, interleukin-8; IL-1β, interleukin-1 beta; TNF-α, tumor necrosis factor alpha; IL-10, interleukin-10; IL-1ra, interleukin-1 receptor antagonist; pre-op, pre-operation; post-op, post-operation; N, number of studies; SMD, standardized mean difference; CI, confidence interval.

Major findings of the meta-analyses are presented in Figs 2 and 3.

#### CRP (peripheral) levels before surgery: In POD

**Fig 4:**
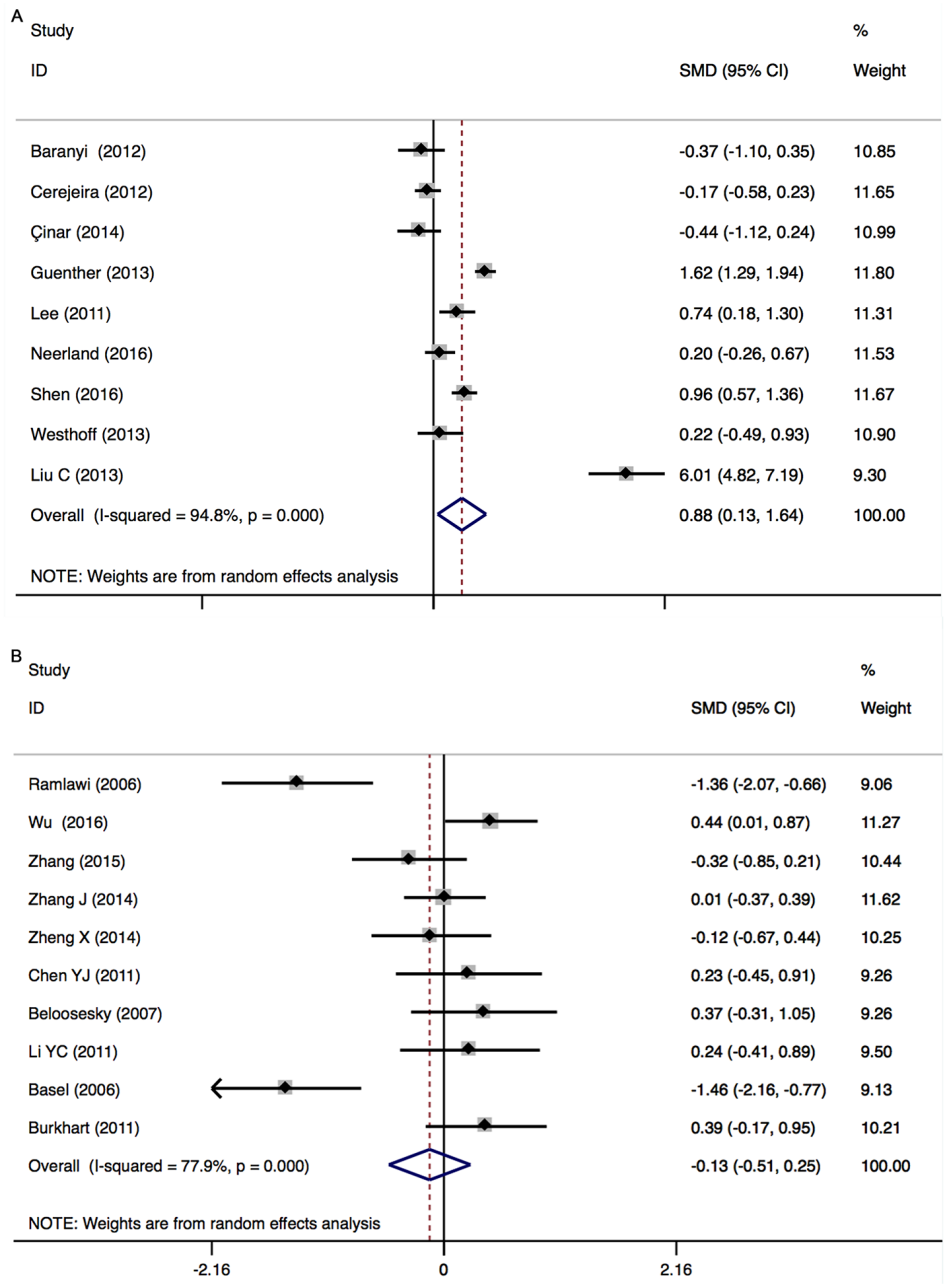
CRP (Peripheral) levels before surgery in patients with POD (A) and POCD (B).

There was a significant increase in peripheral CRP levels in POD patients (9 studies, n[POD/non-POD] = 241/531, SMD 0.883, 95% CI 0.130 to 1.637, *P* = 0.022; REM) with significant heterogeneity between the studies (*P*_*heterogeneity*_ = 0.000; *I*^2^ = 94.8%) (Fig 4A).

#### CRP (peripheral) levels before surgery: In POCD

There was no significant difference in peripheral CRP levels between POCD and non-POCD patients (10 studies, n[POCD/non-POCD] = 211/383, SMD -0.133, 95% CI -0.512 to 0.246, *P* = 0.429; REM) with significant heterogeneity between the studies (*P*_*heterogeneity*_ = 0.000; *I*^2^ = 77.9%) (Fig 4B).

#### IL-6 (peripheral) levels before surgery: In POD

**Fig 5:**
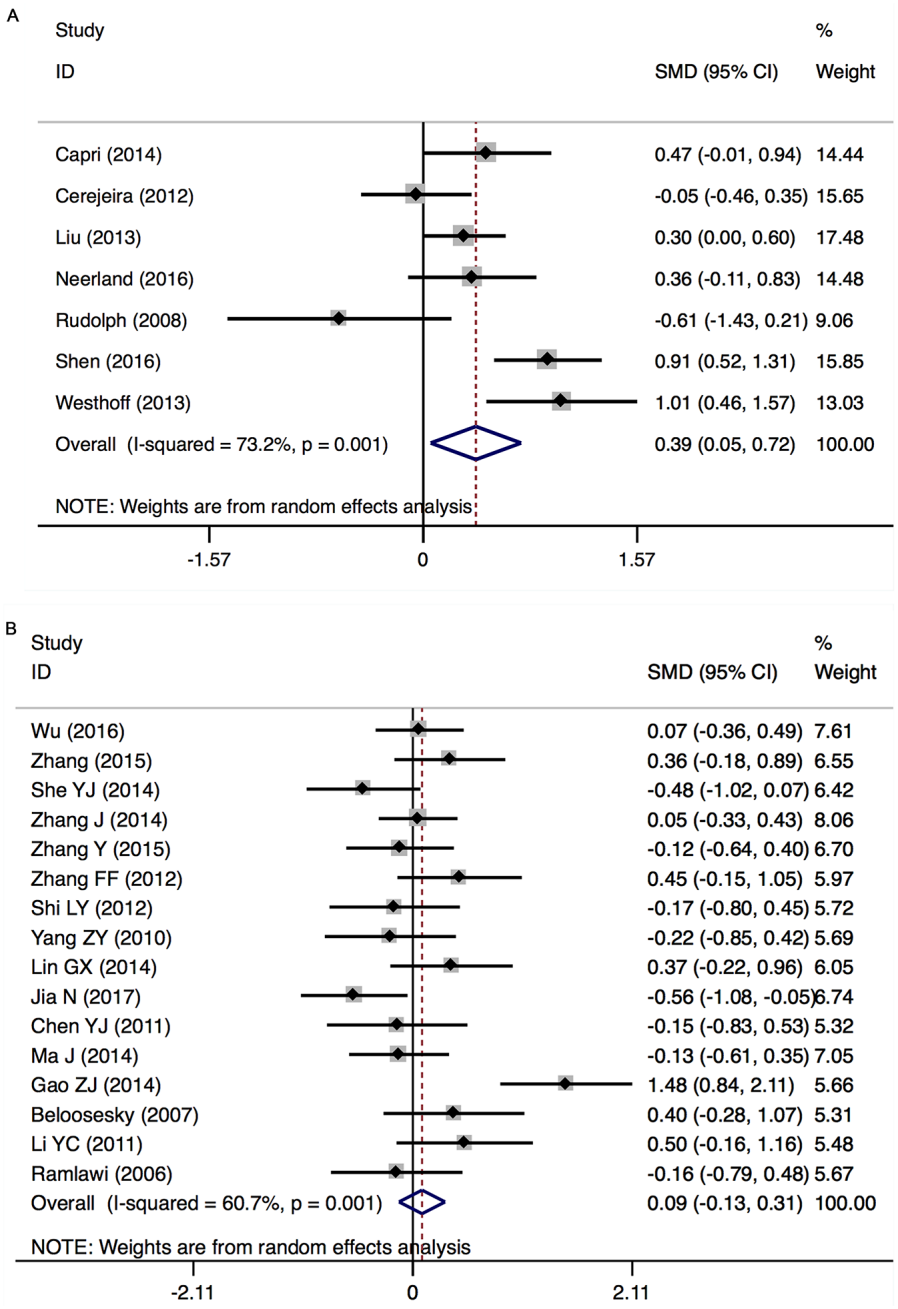
IL-6 (Peripheral) levels before surgery in patients with POD (A) and POCD (B).

There was a significant increase in peripheral IL-6 levels in POD patients (7 studies, n[POD/non-POD] = 241/531, SMD 0.386, 95% CI 0.054 to 0.717, *P* = 0.022; REM) with significant heterogeneity between the studies (*P*_*heterogeneity*_ = 0.001; *I*^2^ = 73.2%) (Fig 5A).

#### IL-6 (peripheral) levels before surgery: In POCD

There was no significant difference in peripheral IL-6 levels between POCD and non-POCD patients (16 studies, n[POCD/non-POCD] = 333/638, SMD 0.089, 95% CI -0.133 to 0.311, *P* = 0.433; REM) with significant heterogeneity between the studies (*P*_*heterogeneity*_ = 0.001; *I*^2^ = 60.7%) (Fig 5B)

#### S-100β (peripheral) levels post-surgery: In POD

**Fig 6:**
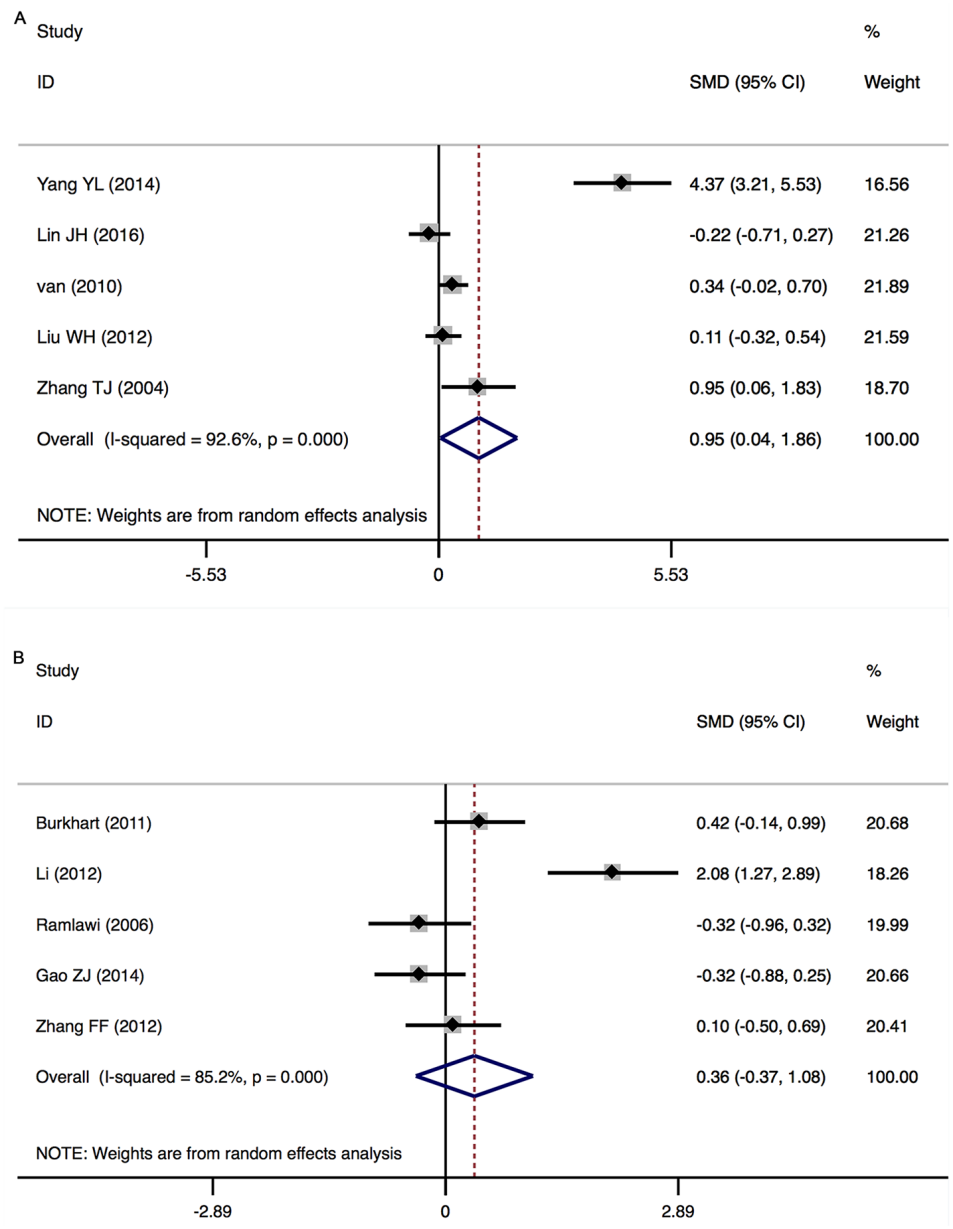
S-100β (Peripheral) Levels Post-Surgery in Patients with POD (A) and POCD (B).

There was a significant increase in peripheral S-100β levels in POD patients (5 studies, n[POD/non-POD] = 151/220, SMD 0.952, 95% CI 0.041 to 1.863, *P* = 0.041; REM) with significant heterogeneity between the studies (*P*_*heterogeneity*_ = 0.000; *I*^2^ = 92.6%) (Fig 6A).

#### S-100β (peripheral) levels post-surgery: In POCD

There was no significant difference in peripheral S-100β levels between POCD and non-POCD patients (5 studies, n[POCD/non-POCD] = 101/120, SMD 0.357, 95% CI -0.368 to 1.083, *P* = 0.334; REM) with significant heterogeneity between the studies (*P*_*heterogeneity*_ = 0.000; *I*^2^ = 85.2%) (Fig 6B).

#### NSE (peripheral) levels before surgery: In POD

There was no significant difference in peripheral NSE levels between POD and non-POD patients (4 studies, n[POCD/non-POCD] = 89/162, SMD 0.139, 95% CI -0.133 to 0.411, *P* = 0.316; FEM) with no heterogeneity between the studies (*P*_*heterogeneity*_ = 0.569; *I*^2^ = 0.0%) (Fig 2).

#### NSE (peripheral) levels before surgery: In POCD

There was a significant increase in peripheral NSE levels in POCD patients (3 studies, n[POD/non-POD] = 65/74, SMD 0.549, 95% CI 0.020 to 1.078, *P* = 0.042; REM) with mild heterogeneity between the studies (*P*_*heterogeneity*_ = 0.096; *I*^2^ = 57.3%) (Fig 3).

#### IL-8 (peripheral) levels after surgery: In POD

Peripheral IL-8 levels significantly increased in the samples taken from POD patients after surgery (3 studies, n[POD/non-POD] = 149/170, SMD 0.243, 95% CI 0.020 to 0.466, *P* = 0.033; FEM) with no heterogeneity between the studies (*P*_*heterogeneity*_ = 0.765; *I*^2^ = 0.0%) (Fig 2).

#### IL-8 (peripheral) levels after surgery: In POCD

No significant differences were found in peripheral IL-8 levels between POCD and control patients (2 studies, n[POD/non-POD] = 29/52, SMD 0.660, 95% CI -0.853 to 2.173, *P* = 0.393; REM; *P*_*heterogeneity*_ = 0.002; *I*^2^ = 89.6%) (Fig 3).

### Subgroup analyses

Above we described the biomarkers that we found to have different effects in POD and POCD perioperatively. CRP and IL-6 levels increased significantly after surgery in patients with POD as well as those with POCD. In addition, we found that preoperative IL-1β, S-100β, IL-8, IL-10, and IL-1ra levels and postoperative TNF-α, and NSE levels did not differ between patients with POD/POCD and normal patients. There are few studies on the relationship between POD/POCD and inflammatory cytokines in CSF [23, 34, 40, 49], and no significant differences were found in the CSF, IL-1ra, IL-6, and CRP levels of patients with and without POD. For the evaluation of other inflammatory cytokines, data were either not available or insufficient for meta-analyses.

**Fig 7:**
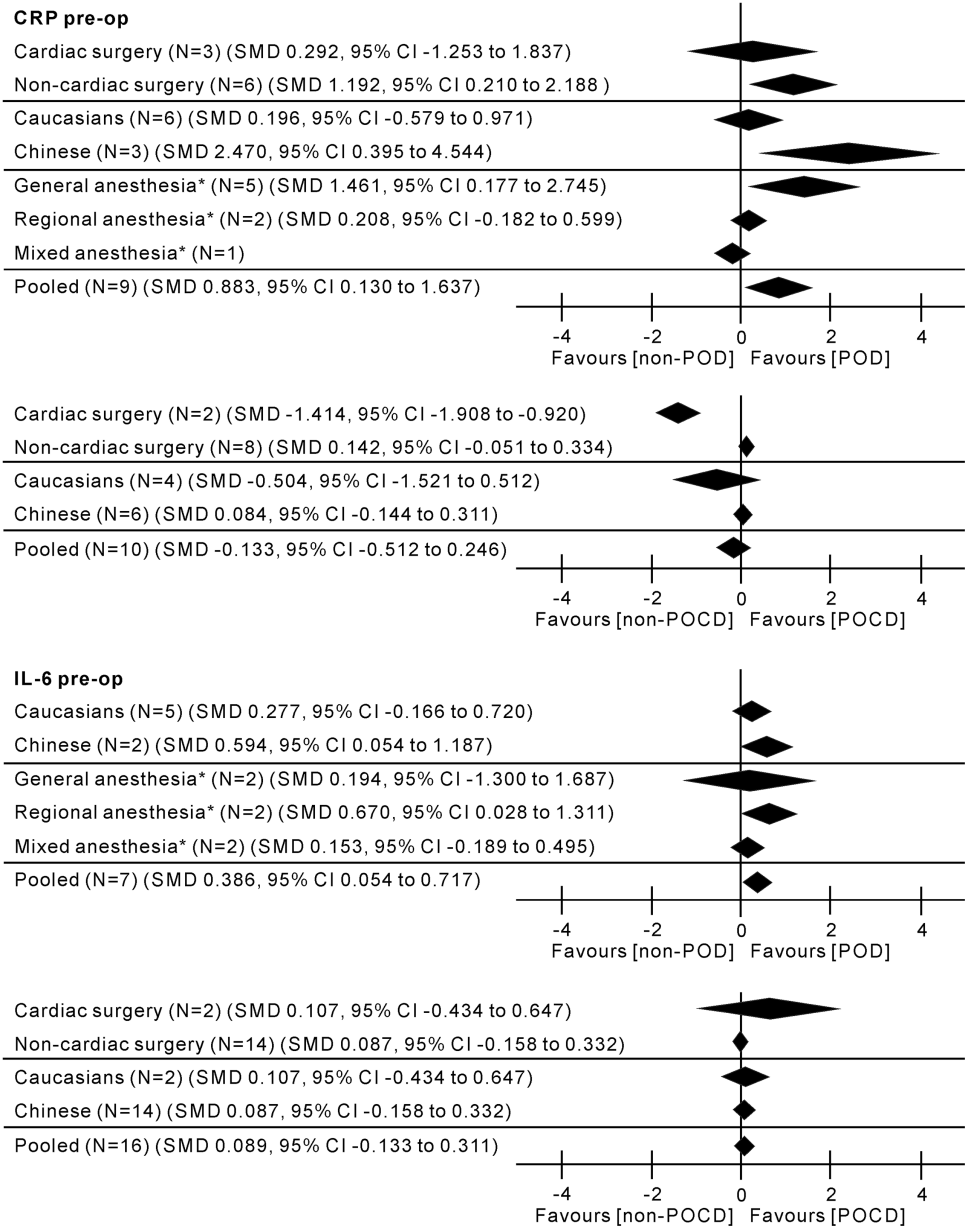
Subgroup analyses for the assessment of impact of surgery type, anesthesia, and ethnicity on peripheral CRP and IL-6 levels perioperatively. Positive values indicate higher levels in POD/POCD patients; negative values indicate higher levels in control patients. All results are from random-effects models. pre-op, pre-operation; post-op, post-operation; N, number of studies; SMD, standardized mean difference; CI, confidence interval; *Data missing for N = 1 study.

In the subgroup analyses, preoperative CRP levels were significantly higher in patients with POD who underwent non-cardiac vs cardiac surgery; general anesthesia vs regional anesthesia; and in Chinese vs Caucasians. Preoperative CRP levels were significantly higher in patients with POCD who underwent cardiac vs non-cardiac surgery; but there were no significant differences between Chinese and Caucasians. Only one study measured IL-6 levels preoperatively in samples from patients with POD who underwent cardiac surgery and there were no differences found between Chinese and Caucasians; but increased significantly in POD patients underwent regional vs general anesthesia. IL-6 levels in patients with POCD did not differ with surgery type or ethnicity (Fig 7 and S3 Table).

### Meta-regression

We conducted meta-regression only if at least ten observations (i.e. ten studies per meta-analysis) were available for each characteristic modelled. In this paper, meta-regression analyses were used to assess the relationships of surgery type and ethnicity with peripheral levels of the studied inflammatory markers. A significant positive association between the SMD of CRP and IL-6 levels from patients with POCD and the surgery type was found (CRP: Coef. = 1.555365, P = 0.001, 10 studies; IL-6: Coef. = -0.6455521, P = 0.086, 16 studies) (Table 1). No associations were found between the SMD and ethnicity (CRP: Coef. = 0.5673341, P = 0.222, 10 studies; IL-6: Coef. = -0.0243988, P = 0.951, 16 studies) (Table 1).

**Table 1:** Meta-regression between the SMDs of peripheral CRP and IL-6 Levels before surgery and the potential sources of heterogeneity.

Potential sources of heterogeneity	Coef.	Std. Err.	t	*P*	95% CI	
CRP levels before surgery in POCD/non-POCD						
Surgery	1.555365	0.2772488	5.61	0.001	0.9160284	2.194702
Ethnicity	0.5673341	0.4286524	1.32	0.222	-0.4211402	1.555808
IL-6 levels before surgery in POCD/non-POCD						
Surgery	-0.6455521	0.3490707	-1.85	0.086	-1.394234	0.1031301
Ethnicity	-0.0243988	0.3926422	-0.06	0.951	-0.8665325	0.8177349

### Sensitivity analysis

Sensitivity analyses were performed to investigate the influence of each individual study on the overall meta-analysis summary estimate and the validity of the effect size. By excluding the included studies one by one, we found that no single study had a significant impact on the outcome of the combined analysis. This suggests that the results of this meta-analysis are stable.

### Publication biases

Significant risk of publication bias was not detected, as demonstrated by funnel plots. Tests for funnel plot asymmetry were only recommended for use when at least 10 studies were included in the meta-analysis. Therefore, Egger’s test was implemented to evaluate asymmetry and publication bias. The results showed no evidence of publication bias (Table 2).

**Table 2:** Egger’s test results for publication and selective reporting bias.

	Egger’s test
Cytokines	*P*
CRP (peripheral) levels before surgery in POD	0.865
CRP (peripheral) levels before surgery in POCD	0.227
IL-6 (peripheral) levels before surgery in POD	0.752
IL-6 (peripheral) levels before surgery in POCD	0.354
S-100β (peripheral) levels post-surgery in POD	0.125
S-100β (peripheral) levels post-surgery in POCD	0.112

## Discussion

Postoperative cognitive impairment is receiving increasing attention, particularly as it primarily affects the elderly population. The fact that POD/POCD is associated with adverse long-term outcomes provides a compelling reason to further investigate the pathogenesis and consequences of POD/POCD. The incidence of POD/POCD varies depending on the study and the type of operation or medical procedure.

There is no formalized medical definition for POD, rather POD falls under the category of postoperative cognitive disorder that is characterized by heightened confusion, a lack of awareness, and a decline in memory and executive function in the brain [77, 78]. According to the ICD-10 Classification of Mental and Behavioural Disorders [79], POD has the following clinical features: The onset of mental status change must be either acute or fluctuating; Delirium is marked by inattention, which is considered the cardinal symptom of delirium [80]; Finally, patients with delirium must have either sleep-wake cycle disturbances, hallucinations, an altered level of consciousness, or disorganized thinking [81].

Without unified diagnostic criteria, POCD is often detected by abnormalities in neuropsychological testing [82, 83]. It may manifest clinically as memory loss, psychomotor derangement, dementia, delirium or depression, difficulties with fine-motor coordination, and impaired higher-level cognitive functions [84, 85]. Several aspects of POCD help to distinguish it from POD [86]. Unlike the early onset of POD (during the initial post-operative period, after an interval of only a few hours or days), POCD usually occurs weeks or sometimes months after the operation. The duration of POD is generally short (1–3 days) whereas POCD can last for several months, sometimes even longer.

The current theories on the etiology of POD/POCD now include surgery-, anesthesia-, and patient-related factors. One factor that is likely to be common to all patients with POD or POCD is an inflammatory state. This meta-analysis aimed to examine and discuss the current published literature concerning delirium and cognitive dysfunction postoperatively, with an emphasis on the evidence for a role of inflammation as a common etiological factor. The current knowledge about the contributing inflammatory biomarkers to POD and POCD his summarized in Figs 2 and 3. POD and POCD have a wide range of contributing mechanisms and some biomarkers overlap.

CRP is a marker of a nonspecific acute-phase response in inflammation, infection, and tissue damage [87], correlated with cognitive decline [88]. An association between high CRP levels and delirium was shown in several studies [89–91]. Patients with POCD have elevated CRP levels after coronary artery bypass grafting [92], liver transplantation [53] and lumbar discectomy [93]. van Munster et al. found elevated levels of IL-6 in patients with delirium [94]. However, in another study the association between delirium and higher IL-6 plasma levels was not confirmed [95]. In this meta-analysis, elevated peripheral CRP and IL-6 levels were detected in both POD and POCD patients postoperatively. We have found that elevated levels of preoperative CRP and IL-6 were associated with POD, but not with POCD.

Likewise, POCD but not POD patients have increased serum levels of NSE preoperatively, which is an indicator of neuronal injury. NSE is a specific enzyme that is mainly present in neurons and neuroendocrine cells and participates in glycolysis [96]. When neurons are damaged or necrotic, NSE enters the CSF and blood. As brain glial cells and other brain tissues do not contain NSE, it is a specific indicator of neuronal damage [97].

As for the other inflammatory markers measured preoperatively, including IL-1β, TNF-α, S-100β, IL-8, IL-10, and IL-1ra, no associations were found between them and POD or POCD. Similarly, there were no effects of postoperative TNF-α or NSE in either POD or POCD. We found that postoperative S-100β and IL-8 levels were associated with POD, while there were few studies concerning their roles in POCD. A few studies in this meta-analysis showed that CRP, IL-6, and IL-1ra levels in the CSF are not connected to POD. However, we cannot draw any exact conclusions as to their role in POCD because of the limited number of studies.

This systematic literature review aimed to identify the current published literature examining inflammatory markers in POD/POCD in order to summarize existing knowledge and to provide a rational basis for future studies. Moreover, the identification of the differences between the inflammatory factors that play a role in POD and POCD advances our understanding of the pathophysiology of POD/POCD and could ultimately lead to new interventions that could improve patient outcomes.

It has been suggested that surgical wounds in combination with anesthetic lesions lead to a primary inflammatory response in the body, which leads to neuroinflammation and the increased risk of developing post-operative cognitive decline [98]. Keeping the importance of neuroinflammation in POCD in mind, targeting these inflammatory processes has been an area of interest for the prevention and treatment of POCD. Statins have been found to exert powerful anti-inflammatory effects and reduce neuroinflammation [99, 100]. Marcela et al. noted an improvement of hippocampal-dependent contextual memory and spatial learning in post-surgical mice which had received atorvastatin, in contrast to post-surgical controls without any treatment [101]. Their findings suggest that the anti-inflammatory and neuroprotective properties of atorvastatin provide a rationale for its use as a therapeutic strategy for postoperative cognitive decline. As for POD, Su et al. demonstrated that the use of a low-dose dexmedetomidine infusion in patients after noncardiac surgery reduced the risk of delirium in the postoperative period [102]. It is the first study to report on the efficacy of a sub-sedative dose of dexmedetomidine to reduce the development of delirium and included patients that were not intubated and those requiring sedation with γ-aminobutyric acid (GABA) receptor agonists. The study adds to the growing body of evidence that dexmedetomidine can reduce the development of delirium [103–105]. Neuroinflammation mediated by microglia has been implicated in delirium. As biomarkers of inflammation were not measured in the abovementioned research, it is not possible to assess whether low-dose dexmedetomidine infusion suppresses the inflammation associated with the aseptic trauma of surgery. Notably, one group found that dexmedetomidine had direct anti-inflammatory effects on microglia, but these were seen only at concentrations an order of magnitude higher than those used clinically [106].

The neuroprotective effects of the drugs mentioned above in animal models and patients are closely related to their anti-inflammatory properties. This confirms that neuroinflammation is closely associated with postoperative cognitive decline, and also suggests that inflammation is an important target for the prevention and treatment of POD/POCD. However, the development of POD/POCD is the result of a comprehensive interaction of various factors. Therefore, it is still necessary to conduct further study on the etiology, molecular mechanisms, and pharmacological interventions of POD/POCD.

There remains the question of the impact of anesthetic technique on POD and POCD. Several clinical trials have attempted to distinguish the effects of regional anesthesia and general anesthesia on POCD. A clinical multi-center evaluation of the impact of anesthesia on POCD was published by Rasmussen and his colleagues [107], which showed a higher incidence of POCD in the general group than the regional one (19.7% vs. 12.5%) one week postoperatively, but no differences were found at 3 months (14.3% vs. 13.9%). Mason et al. concluded that there were no significant differences between general anesthesia and regional or combined anesthesia on the development of POD, but general anesthesia was found to have a slight association with an increased rate of POCD [OR (Odds Ratio) = 1.34, 95% CI 0.93–1.95] [108]. Davis et al. came to a similar conclusion through a meta-analysis of 16 studies, in which three studies showed some difference in cognitive function between regional and general anesthesia, whereas the remaining 13 showed no differences [109]. Fodale et. al believed that the use of certain general anesthetics (e.g. inhalational anesthetics) for elderly patients may worsen amyloid β peptide oligomerization and deposition, thereby increasing the risk of developing POCD [110]. Several studies have shown that the inhalation of small molecular-sized anesthetics do play a leading role in Aβ oligomerization compared to intravenous anesthetics such as propofol or diazepam [111–113]. The specific method of anesthesia was not one of our exclusion or inclusion criteria. As the main concern of this manuscript is to summarize and discuss inflammatory biomarkers in POD/POCD, any study measuring inflammatory marker concentrations in patients with POD/POCD qualified for inclusion. In one case, a study included both patients who received general anesthesia and those who received regional anesthesia. In some other cases, patients who were administered general anesthesia received a combination of both inhalational and intravenous anesthetics. Therefore, we could not address the specific correlation between POD/POCD and the type of anesthesia (e.g. inhalational vs. intravenous, or general vs. regional) in this meta-analysis. Instead, we performed sensitivity analyses to investigate the influence of each individual study on the overall meta-analysis summary estimate and the validity of the effect size.

The main limitation of this study was the small number of papers that could be included in some of the meta-analytical comparisons. As a result, the evidence regarding the roles of IL-1β, TNF-α, IL-8, and NSE in POD/POCD remain inconclusive. The heterogeneity methodology may also have some impact on the overall outcomes; It is possible that our findings cannot be interpreted as truly negative due to the small sample sizes and significant heterogeneity between studies.

Patients with POCD display biomarkers distinct from those with POD, which might be related not only to pathology but also to postoperative time. Most inflammatory markers immediately reached the peak postoperative value [114], but the peak serum concentration was not always the most valuable measure in the prediction of outcome. Therefore, the strength of evidence from this study was undermined by the choice of sampling times that may have missed potential early postoperative differences.

## Conclusion

It is clear that POD and POCD are multifactorial conditions. Among the identified pathomechanisms, some inflammatory biomarkers were common, such as the elevation of CRP and IL-6 postoperatively. S-100β and IL-8 were elevated in only POD postoperatively and during the preoperative period, CRP and IL-6 increased in POD, while NSE levels increased in POCD. No changes in IL-1β, TNF-α, S-100β, IL-8, and IL-10 were found in POD preoperatively, or in TNFα and NSE in POCD postoperatively.

## Supporting information

S1 Checklist Reporting items for the meta-analyses. (DOC)

S1 Appendix Search strategies. (DOCX)

S1 Table Characteristics of the association studies that were included in the meta-analysis and that examined the peripheral and/or the CSF inflammatory markers. (DOCX)

S2 Table Quality score evaluation. (DOCX)

S3 Table Subgroup analyses for the assessment of impact of surgery type, anesthesia, and ethnicity on peripheral CRP and IL-6 levels perioperatively. (DOCX)

## References

[1] Arthur GL, Brende JO, Locicero KA. Diagnostic and statistical manual of mental disorders (4th ed) and text revision. Psychology Psychiatry Autism Family Therapy. 2001.

[2] Maclullich AMJ, Ferguson KJ, Miller T, Rooij SEJAD, Cunningham C. Unravelling the pathophysiology of delirium: A focus on the role of aberrant stress responses. Journal of Psychosomatic Research. 2008;65(3):229 doi: 10.1016/j.jpsychores.2008.05.019 18707945 10.1016/j.jpsychores.2008.05.019 PMC4311661

[3] Young J, Inouye SK. Delirium in older people. Bmj. 2007;334(7598):842–6. doi: 10.1136/bmj.39169.706574.AD 17446616 10.1136/bmj.39169.706574.AD PMC1853193

[4] Inouye SK. Delirium in older persons. N Engl J Med. 2006;354(11):1157–65. doi: 10.1056/NEJMra052321 16540616 10.1056/NEJMra052321

[5] Marcantonio ER. Postoperative Delirium: A 76-Year-Old Woman With Delirium Following Surgery. Jama the Journal of the American Medical Association. 2012;308(1):73 doi: 10.1001/jama.2012.6857 22669559 10.1001/jama.2012.6857 PMC3604975

[6] Md DPSKI, Md PRGJW, Saczynski JS. Delirium in elderly people. 2013.

[7] Lyketsos CG. Prevention of unnecessary hospitalization for patients with dementia: the role of ambulatory care. Jama the Journal of the American Medical Association. 2012;307(2):197–8. doi: 10.1001/jama.2011.2005 22235092 10.1001/jama.2011.2005

[8] Steinmetz J, Christensen KB, Lund T, Lohse N, Rasmussen LS, Group I. Long-term consequences of postoperative cognitive dysfunction. Anesthesiology. 2009;110(3):548 doi: 10.1097/ALN.0b013e318195b569 19225398 10.1097/ALN.0b013e318195b569

[9] Monk TG, Price CC. Postoperative cognitive disorders. Current Opinion in Critical Care. 2011;17(4):376 doi: 10.1097/MCC.0b013e328348bece 21716111 10.1097/MCC.0b013e328348bece PMC3882015

[10] Khadka J, Mcalinden C, Pesudovs K. Cognitive trajectories after postoperative delirium. New England Journal of Medicine. 2012;367(12):1164; author reply 10.1056/NEJMc1209366 22992088

[11] Rudolph JL, Jones RN, Rasmussen LS, Silverstein JH, Inouye SK, Marcantonio ER. Independent vascular and cognitive risk factors for postoperative delirium. American Journal of Medicine. 2007;120(9):807 doi: 10.1016/j.amjmed.2007.02.026 17765051 10.1016/j.amjmed.2007.02.026

[12] Witlox J, Eurelings LS, de Jonghe JF, Kalisvaart KJ, Eikelenboom P, van Gool WA. Delirium in elderly patients and the risk of postdischarge mortality, institutionalization, and dementia: a meta-analysis. Jama. 2010;304(4):443 doi: 10.1001/jama.2010.1013 20664045 10.1001/jama.2010.1013

[13] Association AP. Diagnostic and statistical manual of mental disorders: Text revision: American Psychiatric Association; 2010 4189- p.

[14] Inouye SK, Dyck CHV, Alessi CA, Balkin S, Siegal AP, Horwitz RI. Clarifying Confusion: The Confusion Assessment Method: A New Method for Detection of Delirium. Annals of Internal Medicine. 1990;113(12):941 2240918 10.7326/0003-4819-113-12-941

[15] Trzepacz PT, Baker RW, Greenhouse J. A symptom rating scale for delirium. Psychiatry research. 1988;23(1):89 3363018 10.1016/0165-1781(88)90037-6

[16] Whitfield W. Book Reviews: The ICD10 Classification of Mental and Behavioural Disorders: Clinical Descriptions and Diagnostic Guidelines by World Health Organization. Published by WHO, 1992, 362pp, paperback. ISBN: 92-4-154422-8. Journal of the Royal Society for the Promotion of Health. 1993;113(2):103-.

[17] Folstein MF, Folstein SE, Mchugh PR. Mini-Mental State: A practical method for grading the state of patients for the clinician. Journal of Psychiatric Research. 1975;12(3):189–98. 1202204 10.1016/0022-3956(75)90026-6

[18] Hozo SP, Djulbegovic B, Hozo I. Estimating the mean and variance from the median, range, and the size of a sample. Bmc Medical Research Methodology. 2005;5(1): 13. 15840177 10.1186/1471-2288-5-13 PMC1097734

[19] Higgins JPT, White IR, Anzures-Cabrera J. Meta-analysis of skewed data: Combining results reported on log-transformed or raw scales. Statistics in Medicine. 2008;27(29):6072 doi: 10.1002/sim.3427 18800342 10.1002/sim.3427 PMC2978323

[20] Wells GA, Shea BJ, O'Connell D, Peterson J, Welch V, Losos M, et al The Newcastle–Ottawa Scale (NOS) for Assessing the Quality of Non-Randomized Studies in Meta-Analysis. Applied Engineering in Agriculture. 2014;18(6):págs. 727–34.

[21] Baranyi A, Rothenhaeusler H-B. The impact of intra- and postoperative albumin levels as a biomarker of delirium after cardiopulmonary bypass: Results of an exploratory study. Psychiatry Research. 2012;200(2–3):957–63. doi: 10.1016/j.psychres.2012.05.030 22749153 10.1016/j.psychres.2012.05.030

[22] Burkhart CS, Dell-Kuster S, Gamberini M, Moeckli A, Grapow M, Filipovic M, et al Modifiable and Nonmodifiable Risk Factors for Postoperative Delirium After Cardiac Surgery With Cardiopulmonary Bypass. Journal of Cardiothoracic and Vascular Anesthesia. 2010;24(4):555–9. doi: 10.1053/j.jvca.2010.01.003 20227891 10.1053/j.jvca.2010.01.003

[23] Cape E, Hall RJ, van Munster BC, de Vries A, Howie SEM, Pearson A, et al Cerebrospinal fluid markers of neuroinflammation in delirium: A role for interleukin-1β in delirium after hip fracture. Journal of Psychosomatic Research. 2014;77(3):219–25. doi: 10.1016/j.jpsychores.2014.06.014 25124807 10.1016/j.jpsychores.2014.06.014 PMC4274366

[24] Capri M, Yani SL, Chattat R, Fortuna D, Bucci L, Lanzarini C, et al Preoperative, high IL-6 blood level is a risk factor of postoperative delirium onset in old patients. Frontiers in Endocrinology. 2014;5(SEP). doi: 10.3389/fendo.2014.00173 25368603 10.3389/fendo.2014.00173 PMC4201145

[25] Cerejeira J, Batista P, Nogueira V, Vaz-Serra A, Mukaetova-Ladinska EB. The stress response to surgery and postoperative delirium: Evidence of hypothalamic-pituitary-adrenal axis hyperresponsiveness and decreased suppression of the GH/IGF-1 axis. Journal of Geriatric Psychiatry and Neurology. 2013;26(3):185–94. doi: 10.1177/0891988713495449 23864592 10.1177/0891988713495449

[26] Cerejeira JMS, Nogueira V, Luís P, Vaz-Serra A, Mukaetova-Ladinska EB. The cholinergic system and inflammation: Common pathways in delirium pathophysiology. Journal of the American Geriatrics Society. 2012;60(4):669–75. doi: 10.1111/j.1532-5415.2011.03883.x 22316182 10.1111/j.1532-5415.2011.03883.x

[27] Chu C-S, Liang C-K, Chou M-Y, Lin Y-T, Hsu C-J, Chu C-L, et al Lack of Association between Pre-Operative Insulin-Like Growth Factor-1 and the Risk of Post-Operative Delirium in Elderly Chinese Patients. Psychiatry Investigation. 2016;13(3):327–32. doi: 10.4306/pi.2016.13.3.327 27247600 10.4306/pi.2016.13.3.327 PMC4878968

[28] Çinar MA, Balikçi A, Sertoğlu E, Ak M, Serdar MA, Özmenler KN. Role of CRP, TNF-α and IGF-1 in delirium athophysiology. Noropsikiyatri Arsivi. 2014;51(4):376–82. doi: 10.5152/npa.2014.6999 28360657 10.5152/npa.2014.6999 PMC5353173

[29] Guenther U, Theuerkauf N, Frommann I, Brimmers K, Malik R, Stori S, et al Predisposing and precipitating factors of delirium after cardiac surgery: A prospective observational cohort study. Annals of Surgery. 2013;257(6):1160–7. doi: 10.1097/SLA.0b013e318281b01c 23426334 10.1097/SLA.0b013e318281b01c

[30] Kazmierski J, Banys A, Latek J, Bourke J, Jaszewski R. Cortisol levels and neuropsychiatric diagnosis as markers of postoperative delirium: A prospective cohort study. Critical Care. 2013;17(2). doi: 10.1186/cc12548 23452669 10.1186/cc12548 PMC3733427

[31] Kazmierski J, Banys A, Latek J, Bourke J, Jaszewski R. Raised IL-2 and TNF-α concentrations are associated with postoperative delirium in patients undergoing coronary-artery bypass graft surgery. International psychogeriatrics / IPA. 2014;26(5):845–55. doi: 10.1017/S1041610213002378 24345656 10.1017/S1041610213002378

[32] Lee HJ, Hwang DS, Wang SK, Ghee IS, Baeg S, Kim JL. Early Assessment of Delirium in Elderly Patients after Hip Surgery. Psychiatry Investigation. 2011;8(4):340–7. doi: 10.4306/pi.2011.8.4.340 22216044 10.4306/pi.2011.8.4.340 PMC3246142

[33] Liu P, Li YW, Wang XS, Zou X, Zhang DZ, Wang DX, et al High serum interleukin-6 level is associated with increased risk of delirium in elderly patients after noncardiac surgery: A prospective cohort study. Chinese Medical Journal. 2013;126(19):3621–7. 24112153

[34] Neerland BE, Hall RJ, Seljeflot I, Frihagen F, MacLullich AMJ, Ræder J, et al Associations Between Delirium and Preoperative Cerebrospinal Fluid C-Reactive Protein, Interleukin-6, and Interleukin-6 Receptor in Individuals with Acute Hip Fracture. Journal of the American Geriatrics Society. 2016;64(7):1456–63. doi: 10.1111/jgs.14238 27341529 10.1111/jgs.14238

[35] Plaschke K, Fichtenkamm P, Schramm C, Hauth S, Martin E, Verch M, et al Early postoperative delirium after open-heart cardiac surgery is associated with decreased bispectral EEG and increased cortisol and interleukin-6. Intensive Care Medicine. 2010;36(12):2081–9. doi: 10.1007/s00134-010-2004-4 20689917 10.1007/s00134-010-2004-4

[36] Rudolph JL, Ramlawi B, Kuchel GA, McElhaney JE, Xie D, Sellke FW, et al Chemokines are associated with delirium after cardiac surgery. The journals of gerontology Series A, Biological sciences and medical sciences\. 2008;63\(2\):184–9\. Epub 2008/03/04. \. 18314455 10.1093/gerona/63.2.184 PMC2735245

[37] Shen H, Shao Y, Chen J, Guo J. Insulin-like growth factor-1, a potential predicative biomarker for postoperative delirium among elderly patients with open abdominal surgery. Current Pharmaceutical Design. 2016;22(38):5879–83. doi: 10.2174/1381612822666160813234311 27526790 10.2174/1381612822666160813234311

[38] van Munster BC, Bisschop PH, Zwinderman AH, Korevaar JC, Endert E, Wiersinga WJ, et al Cortisol, interleukins and S100B in delirium in the elderly. Brain and Cognition. 2010;74(1):18–23. doi: 10.1016/j.bandc.2010.05.010 20580479 10.1016/j.bandc.2010.05.010

[39] van Munster BC, Korevaar JC, Zwinderman AH, Levi M, Wiersinga WJ, De Rooij SE. Time-course of cytokines during delirium in elderly patients with hip fractures. Journal of the American Geriatrics Society\. 2008;56\(9\):1704–9\. Epub 2008/08/12. doi: 10.1111/j.1532-5415.2008.01851.x \. 18691278 10.1111/j.1532-5415.2008.01851.x

[40] Westhoff D, Witlox J, Koenderman L, Kalisvaart KJ, de Jonghe JFM, van Stijn MFM, et al Preoperative cerebrospinal fluid cytokine levels and the risk of postoperative delirium in elderly hip fracture patients. Journal of Neuroinflammation. 2013;10 doi: 10.1186/1742-2094-10-122 24093540 10.1186/1742-2094-10-122 PMC3851488

[41] Yen TE, Allen JC, Rivelli SK, Patterson SC, Metcalf MR, Flink BJ, et al Association between Serum IGF-I levels and Postoperative Delirium in Elderly Subjects Undergoing Elective Knee Arthroplasty. Scientific Reports. 2016;6 doi: 10.1038/srep20736 26846868 10.1038/srep20736 PMC4742946

[42] Ren Q. Clinical investigation and animal model exploration of postoperative delirium [PhD thesis]: DongNan University; 2015.

[43] Liu W, Du L, Wang T, Yang J, Li H. The Relationships of Emergence Delirium and S100β, NSE. Progress in Modern Biomedicine. 2012;12(22):4298–300.

[44] Liu C. Preliminary Clinical Study of Delirium after Endovascular Exclusion for Aortic Dissection [thesis]: Zhangzhou University; 2013.

[45] Zhang T. Clinical and experimental study of mental disorders after cardiopulmonary bypass in elderly patients [thesis]: Shanghai Jiaotong Univwersity; 2004.

[46] Yang Y, Ma W, Zhang C, Li Z, Zhao Z. Change and Significience of Plasma S-100β Protein and NSE in Elderly Patients with Delirium after Hip Joint Replacement. Journal of Kunming Medical University. 2014;(04):61–4.

[47] Lin J, Zhou G, Yue Y, Wu A. Relation among brain-derived neurotrophic factor, neuron specific enolase, Sl∞B protein and postoperative delirium in elderly patienisVIritll hip joint operation. China Medicine. 2016;11(7):1055–8.

[48] Jiang H, Ma M, Lao M. Research on the relationship between C reactive protein and postoperative delirium of elderly patients after hip fracture. Chin J Prim Med Pharm. 2014;(19):2944–5.

[49] Chen M, Gao H, Huang L, Jin J. The correlation between acute stage delirium and cerebrospinal fluid neuronal specific enolase and lactic acid levels in the postoperative acute stage of hip fracture. Zhejiang Journal of Traumatic Surgery. 2016;(01):104–6.

[50] Sun L, Jia P, Zhang J, Zhang X, Zhang Y, Jiang H, et al Production of inflammatory cytokines, cortisol, and Aβ1–40 in elderly oral cancer patients with postoperative delirium. Neuropsychiatric Disease and Treatment. 2016;12:2789–95. doi: 10.2147/NDT.S113077 27822051 10.2147/NDT.S113077 PMC5089834

[51] Goettel N, Burkhart CS, Rossi A, Cabella BCT, Berres M, Monsch AU, et al Associations between Impaired Cerebral Blood Flow Autoregulation, Cerebral Oxygenation, and Biomarkers of Brain Injury and Postoperative Cognitive Dysfunction in Elderly Patients after Major Noncardiac Surgery. Anesthesia and Analgesia. 2017;124(3):934–42. doi: 10.1213/ANE.0000000000001803 28151820 10.1213/ANE.0000000000001803

[52] Burkhart CS, Rossi A, Dell-Kuster S, Strebel SP, Monsch AU, Steiner LA. Postoperative cognitive dysfunction (POCD), markers of brain damage and systemic inflammation in elderly patients. European Journal of Anaesthesiology. 2011;28:223.

[53] Li X, Wen D-X, Zhao Y-H, Hang Y-N, Mandell MS. Increase of beta-amyloid and C-reactive protein in liver transplant recipients with postoperative cognitive dysfunction. Hepatobiliary & Pancreatic Diseases International. 2013;12(4):370–6. doi: 10.1016/S1499-3872(13)60058-2 23924494 10.1016/s1499-3872(13)60058-2

[54] Li YC, Xi CH, An YF, Dong WH, Zhou M. Perioperative inflammatory response and protein S-100 beta concentrations—relationship with post-operative cognitive dysfunction in elderly patients. Acta Anaesthesiologica Scandinavica. 2012;56(5):595–600. doi: 10.1111/j.1399-6576.2011.02616.x 22224444 10.1111/j.1399-6576.2011.02616.x

[55] Lin GX, Wang T, Chen MH, Hu ZH, Ouyang W. Serum high-mobility group box 1 protein correlates with cognitive decline after gastrointestinal surgery. Acta Anaesthesiologica Scandinavica. 2014;58(6):668–74. doi: 10.1111/aas.12320 24754551 10.1111/aas.12320

[56] Ramlawi B, Rudolph JL, Mieno S, Khabbaz K, Sodha NR, Boodhwani M, et al Serologic markers of brain injury and cognitive function after cardiopulmonary bypass. Annals of Surgery. 2006;244(4):593–600. doi: 10.1097/01.sla.0000239087.00826.b4 16998368 10.1097/01.sla.0000239087.00826.b4 PMC1856569

[57] Wu C, Wang R, Li X, Chen J. Preoperative Serum MicroRNA-155 Expression Independently Predicts Postoperative\ Cognitive Dysfunction After Laparoscopic Surgery for Colon Cancer. Medical science monitor: international medical journal of experimental and\ clinical research\. 2016;22\:4503–8\. Epub 2016/11/23. 27872469 10.12659/MSM.898397 PMC5123778

[58] She Y, Dong L, Xing C, Huo J, Zhou Z, Yu Y. Changes of serum cytokines in patients with cognitive dysfunction after hip arthroplasty. Jiangsu Med J. 2014;(02):234–5.

[59] Zhang J, Chen L, Wang W. The relationship between perioperative inflammatory response and postoperative cognitive function in elderly hyperglycemia patients. Chinese Journal of Gerontology. 2014;(18):5097–9.

[60] Zhang Y, Qian Y, Si Y, Bao H, Zhou J. Differential expressions of serum cytokines in cognitive dysfunction patients after colorectal surgery. Chin J Cell Mol Immunol. 2015;(02):231–4. 25652866

[61] Zhang F, Zhang J, Yang L. Relativity Rsearch of Expression of S-100β Protein, IL-1β, IL-6 and α-TNF to Recognition after Total Knee Arthroplasty. Journal of Zhejiang University of Traditional Chinese Medicine. 2012;(12):1290–2.

[62] Shi L, Xu J, Wan Y. Relationship between postoperative cognitive dysfunction and the expression of inflammatory cytokines of plasma. Shanghai Medical Journal. 2012;(02):115–7.

[63] Yang Z, Xu Y, Fei F, Lv H, Li Q, Yang L, et al Postoperative cognitive dysfunction and expression of IL-1β, IL-6 and TNF-α in the elderly. J Clin Anesthesial 2010;(09):764–6.

[64] Jia N, Ma Y, Gu X. Changes of cognitive function and inflammatory factor level in elderly patients with lower limb orthopaedic surgery. Chinese Journal of Gerontology. 2017;(02):431–3.

[65] Zheng X, Peng H, Ma Z, Gu X. The relationship between early postoperative cognitive dysfunction and the concentration of plasma c-reactive protein in the perioperative stage of adolescent idiopathic scoliosis. China Journal of Modern Medicine. 2014;(26):36–40.

[66] Chen Y, Huang C, Gong J, Lu Z, Yang Z, Zhou H. Relationship between hs-CRP, IL-6 and the cognitive function decrease of elderly after sevoflurane anesthesia. Chinese journal of Health Labratory Technology. 2011;(10):2431–3.

[67] Ma J, Chen Y, Zhang J. Reduced platelet APPr and altered inflammatory factors in postoperative cognitive dysfunction of elderly. J Trop Med 2014;(07):874–6+80.

[68] Gao Z. Exploration of neuroimaging and biochemical prognostic indicators for postoperative cognitive dysfunction after coronary artery bypass graft [thesis]: The Fourth Military Medical University; 2014.

[69] Beloosesky Y, Hendel D, Weiss A, Hershkovitz A, Grinblat J, Pirotsky A, et al Cytokines and C-reactive protein production in hip-fracture-operated elderly\ patients. The journals of gerontology Series A, Biological sciences and medical sciences\. 2007;62\(4\):420–6\. Epub 2007/04/25. \. 17452737 10.1093/gerona/62.4.420

[70] Ramlawi B, Rudolph JL, Mieno S, Feng J, Boodhwani M, Khabbaz K, et al C-Reactive protein and inflammatory response associated to neurocognitive decline following cardiac surgery. Surgery. 2006;140(2):221–6. doi: 10.1016/j.surg.2006.03.007 16904973 10.1016/j.surg.2006.03.007

[71] Hu X, Xie H, Zhou G, Guo Je, Ni X. Relationship between cognitive dysfunction and plasma cortisol levels in elderly patients after hip replacement. Journal of New Medicine. 2015;(07):448–52.

[72] Li Y, Fan A, Dong W, Zhou M. Correlation between postoperative cognitive dysfunction and peri-operative inflammation in elderly patients undergoing total hip-replacement surgery. Shanghai Medical Journal. 2011;34(4):249–52.

[73] Zhang YH, Guo XH, Zhang QM, Yan GT, Wang TL. Serum CRP and urinary trypsin inhibitor implicate postoperative cognitive dysfunction especially in elderly patients. International Journal of Neuroscience. 2015;125(7):501–6. doi: 10.3109/00207454.2014.949341 25105909 10.3109/00207454.2014.949341

[74] Zhang Q. Correlation between Blood Pressure Variability and Postoperative Delirium and Postoperative Cognitive Dysfunction in Elderly Patients Undergoing Non-cardiac Surgery [thesis]: Xinan Medical University; 2016.

[75] Wei LA, Fearing MA, Sternberg EJ, Inouye SK. The Confusion Assessment Method: a systematic review of current usage. J Am Geriatr Soc. 2008;56(5):823–30. doi: 10.1111/j.1532-5415.2008.01674.x 18384586 10.1111/j.1532-5415.2008.01674.x PMC2585541

[76] Rasmussen LS, Larsen K, Houx P, Skovgaard LT, Hanning CD, Moller JT. The assessment of postoperative cognitive function. Acta Anaesthesiologica Scandinavica. 2001;45(3):275–89. 11207462 10.1034/j.1399-6576.2001.045003275.x

[77] Nelson JE, Tandon NMercado AF, Camhi SL, Ely EW, Morrison RS. Brain dysfunction: another burden for the chronically critically ill. Archives of Internal Medicine. 2006;166(18):1993–9. doi: 10.1001/archinte.166.18.1993 17030833 10.1001/archinte.166.18.1993

[78] Monk TG, Price CC. Postoperative cognitive disorders. Current Opinion in Critical Care. 2011;17(4):376–81. doi: 10.1097/MCC.0b013e328348bece 21716111 10.1097/MCC.0b013e328348bece PMC3882015

[79] Organization WH. The ICD-10 classification of mental and behavioural disorders: clinical descriptions and diagnostic guidelines. Geneva World Health Organization. 1993;10(2):86–92.

[80] Blazer DG, van Nieuwenhuizen AO. Evidence for the diagnostic criteria of delirium: an update. Curr Opin Psychiatry. 2012;25(3):239–43. doi: 10.1097/YCO.0b013e3283523ce8 22449764 10.1097/YCO.0b013e3283523ce8

[81] Ely EW, Inouye SK, Bernard GR, Gordon S, Francis J, May L, et al Delirium in mechanically ventilated patients: validity and reliability of the confusion assessment method for the intensive care unit (CAM-ICU). JAMA. 2001;286(21):2703–10. 11730446 10.1001/jama.286.21.2703

[82] Seymour DG, Severn AM. Cognitive dysfunction after surgery and anaesthesia: what can we tell the grandparents? Age Ageing. 2009;38(2):147–50. doi: 10.1093/ageing/afn289 19153069 10.1093/ageing/afn289

[83] Mrak RE, Griffin ST, Graham DI. Aging-associated changes in human brain. J Neuropathol Exp Neurol. 1997;56(12):1269–75. 9413275 10.1097/00005072-199712000-00001

[84] Raja PV, Blumenthal JA, Doraiswamy PM. Cognitive deficits following coronary artery bypass grafting: prevalence, prognosis, and therapeutic strategies. CNS Spectr. 2004;9(10):763–72. 15448586 10.1017/s1092852900022409

[85] Butterfield NN, Graf P, Ries CR, MacLeod BA. The effect of repeated isoflurane anesthesia on spatial and psychomotor performance in young and aged mice. Anesth Analg. 2004;98(5):1305–11, table of contents. 15105206 10.1213/01.ane.0000108484.91089.13

[86] Nadelson MR, Sanders RD, Avidan MS. Perioperative cognitive trajectory in adults. Br J Anaesth. 2014;112(3):440–51. doi: 10.1093/bja/aet420 24384981 10.1093/bja/aet420

[87] Pepys MB, Hirschfield GM. Pepys MB, Hirschfield GM. C-reactive protein: a critical update. J Clin Invest 111, 1805–1812. 2003;111(12):1805–12. 10.1172/JCI18921 PMC161431 12813013

[88] Tilvis RS, Kahonen-Vare MH, Jolkkonen J, Valvanne J, Pitkala KH, Strandberg TE. Predictors of cognitive decline and mortality of aged people over a 10-year period. J Gerontol A Biol Sci Med Sci. 2004;59(3):268–74. 15031312 10.1093/gerona/59.3.m268

[89] Beloosesky Y, Grinblat J, Pirotsky A, Weiss A, Hendel D. Different C-Reactive Protein Kinetics in Post-Operative Hip-Fractured Geriatric Patients with and without Complications. Gerontology. 2004;50(4):216–22. doi: 10.1159/000078350 15258426 10.1159/000078350

[90] Burkhart CS, Dell-Kuster S, Gamberini M, Moeckli A, Grapow M, Filipovic M, et al Modifiable and nonmodifiable risk factors for postoperative delirium after cardiac surgery with cardiopulmonary bypass. Journal of Cardiothoracic & Vascular Anesthesia. 2010;24(4):555. 20227891 10.1053/j.jvca.2010.01.003

[91] Zhang Z, Pan L, Deng H, Ni H, Xu X. Prediction of delirium in critically ill patients with elevated C-reactive protein. Journal of Critical Care. 2014;29(1):88–92. doi: 10.1016/j.jcrc.2013.09.002 24120090 10.1016/j.jcrc.2013.09.002

[92] Hudetz JA, Gandhi SD, Iqbal Z, Patterson KM, Pagel PS. Elevated postoperative inflammatory biomarkers are associated with short- and medium-term cognitive dysfunction after coronary artery surgery. Journal of Anesthesia. 2011;25(1):1–9. doi: 10.1007/s00540-010-1042-y 21061037 10.1007/s00540-010-1042-y

[93] Zhang YH, Guo XH, Zhang QM, Yan GT, Wang TL. Serum CRP and urinary trypsin inhibitor implicate postoperative cognitive dysfunction especially in elderly patients. Int J Neurosci. 2015;125(7):501–6. doi: 10.3109/00207454.2014.949341 25105909 10.3109/00207454.2014.949341

[94] van Munster BC, Zwinderman AH, de Rooij SE. Genetic variations in the interleukin-6 and interleukin-8 genes and the interleukin-6 receptor gene in delirium. Rejuvenation Res. 2011;14(4):425–8. doi: 10.1089/rej.2011.1155 21851175 10.1089/rej.2011.1155

[95] Adamis D, Treloar A, Martin FC, Gregson N, Hamilton G, Macdonald AJ. APOE and cytokines as biological markers for recovery of prevalent delirium in elderly medical inpatients. Int J Geriatr Psychiatry. 2007;22(7):688–94. doi: 10.1002/gps.1732 17203511 10.1002/gps.1732

[96] Lipowski ZJ. Current concepts—geriatrics: Delirium in the elderly patient. New England Journal of Medicine. 1989:578–82. doi: 10.1056/NEJM198903023200907 2644535 10.1056/NEJM198903023200907

[97] Fan X, Lei P. Changes of NSE and NGF in serum after craniocerebral injury and its clinical significance. Progress in Modern Biomedicine. 2011;(06):1191–3.

[98] Kapila AK, Watts HR, Wang T, Ma D. The impact of surgery and anesthesia on post-operative cognitive decline and\ Alzheimer's disease development: biomarkers and preventive strategies. Journal of Alzheimer's disease: JAD\. 2014;41\(1\):1–13\. Epub 2014/03/01. doi: 10.3233/JAD-132258 \. 24577482 10.3233/JAD-132258

[99] Clarke RM, Lyons A, O'Connell F, Deighan BF, Barry CE, Anyakoha NG, et al A pivotal role for interleukin-4 in atorvastatin-associated neuroprotection in rat brain. J Biol Chem. 2008;283(4):1808–17. doi: 10.1074/jbc.M707442200 17981803 10.1074/jbc.M707442200

[100] Pac-Soo C, Lloyd DG, Vizcaychipi MP, Ma D. Statins: the role in the treatment and prevention of Alzheimer's neurodegeneration. J Alzheimers Dis. 2011;27(1):1–10. doi: 10.3233/JAD-2011-110524 21734347 10.3233/JAD-2011-110524

[101] Vizcaychipi MP, Watts HR, O'Dea KP, Lloyd DG, Penn JW, Wan Y, et al The therapeutic potential of atorvastatin in a mouse model of postoperative\ cognitive decline. Annals of surgery\. 2014;259\(6\):1235–44\. Epub 2013/11/23. doi: 10.1097/SLA.0000000000000257 \. 24263322 10.1097/SLA.0000000000000257

[102] Su X, Meng Z-T, Wu X-H, Cui F, Li H-L, Wang D-X, et al Dexmedetomidine for prevention of delirium in elderly patients after non-cardiac surgery: a randomised, double-blind, placebo-controlled trial. The Lancet. 2016;388(10054):1893–902. https://doi.org/10.1016/S0140-6736(16)30580-3. 10.1016/S0140-6736(16)30580-3 27542303

[103] Pandharipande PP, Pun BT, Herr DL, Maze M, Girard TD, Miller RR, et al Effect of sedation with dexmedetomidine vs lorazepam on acute brain dysfunction in mechanically ventilated patients: the MENDS randomized controlled trial. JAMA. 2007;298(22):2644–53. doi: 10.1001/jama.298.22.2644 18073360 10.1001/jama.298.22.2644

[104] Riker RR, Shehabi Y, Bokesch PM, Ceraso D, Wisemandle W, Koura F, et al Dexmedetomidine vs midazolam for sedation of critically ill patients: a randomized trial. JAMA. 2009;301(5):489–99. doi: 10.1001/jama.2009.56 19188334 10.1001/jama.2009.56

[105] Djaiani G, Silverton N, Fedorko L, Carroll J, Styra R, Rao V, et al Dexmedetomidine versus Propofol Sedation Reduces Delirium after Cardiac Surgery: A Randomized Controlled Trial. Anesthesiology. 2016;124(2):362–8. doi: 10.1097/ALN.0000000000000951 26575144 10.1097/ALN.0000000000000951

[106] Peng M, Wang YL, Wang CY, Chen C. Dexmedetomidine attenuates lipopolysaccharide-induced proinflammatory response in primary microglia. J Surg Res. 2013;179(1):e219–25. doi: 10.1016/j.jss.2012.05.047 22683080 10.1016/j.jss.2012.05.047

[107] Rasmussen LS, Johnson T, Kuipers HM, Kristensen D, Siersma VD, Vila P, et al Does anaesthesia cause postoperative cognitive dysfunction? A randomised study of regional versus general anaesthesia in 438 elderly patients. Acta Anaesthesiol Scand. 2003;47(3):260–6. 12648190 10.1034/j.1399-6576.2003.00057.x

[108] Mason SE, Noel-Storr A, Ritchie CW. The impact of general and regional anesthesia on the incidence of post-operative cognitive dysfunction and post-operative delirium: a systematic review with meta-analysis. J Alzheimers Dis. 2010;22 Suppl 3:67–79. doi: 10.3233/JAD-2010-101086 20858956 10.3233/JAD-2010-101086

[109] Davis N, Lee M, Lin AY, Lynch L, Monteleone M, Falzon L, et al Postoperative cognitive function following general versus regional anesthesia: a systematic review. J Neurosurg Anesthesiol. 2014;26(4):369–76. doi: 10.1097/ANA.0000000000000120 25144505 10.1097/ANA.0000000000000120 PMC4156882

[110] Fodale V, Santamaria LB, Schifilliti D, Mandal PK. Anaesthetics and postoperative cognitive dysfunction: a pathological mechanism mimicking Alzheimer's disease. Anaesthesia. 2010;65(4):388–95. doi: 10.1111/j.1365-2044.2010.06244.x 20136805 10.1111/j.1365-2044.2010.06244.x

[111] Mandal PK, Simplaceanu V, Fodale V. Intravenous anesthetic diazepam does not induce amyloid-beta peptide oligomerization but diazepam co-administered with halothane oligomerizes amyloid-beta peptide: an NMR study. J Alzheimers Dis. 2010;20(1):127–34. doi: 10.3233/JAD-2010-1350 20164598 10.3233/JAD-2010-1350

[112] Mandal PK, Bhavesh NS, Chauhan VS, Fodale V. NMR investigations of amyloid-beta peptide interactions with propofol at clinically relevant concentrations with and without aqueous halothane solution. J Alzheimers Dis. 2010;21(4):1303–9. 21504123 10.3233/jad-2010-100396

[113] Mandal PK, Ahuja M. Comprehensive nuclear magnetic resonance studies on interactions of amyloid-beta with different molecular sized anesthetics. J Alzheimers Dis. 2010;22 Suppl 3:27–34. doi: 10.3233/JAD-2010-101128 20858952 10.3233/JAD-2010-101128

[114] Terrando N, Monaco C, Ma D, Foxwell BM, Feldmann M, Maze M. Tumor necrosis factor-alpha triggers a cytokine cascade yielding postoperative cognitive decline. Proc Natl Acad Sci U S A. 2010;107(47):20518–22. doi: 10.1073/pnas.1014557107 21041647 10.1073/pnas.1014557107 PMC2996666

