# Correction: Inflammatory markers in postoperative delirium (POD) and cognitive dysfunction (POCD): A meta-analysis of observational studies

**DOI:** 10.1371/journal.pone.0209284

**Published:** 2018-12-13

**Authors:** Xuling Liu, Yang Yu, Shengmei Zhu

There is an error in the penultimate sentence of the Abstract. The correct sentence is: Available evidence from medium-to-high quality observational studies suggests that POD and POCD are indeed correlated with the concentration of peripheral inflammatory markers.
