# Supplementary material for: Inflammatory markers in postoperative delirium (POD) and cognitive dysfunction (POCD): A meta-analysis of observational studies
Source: PLoS One. 2018 Apr 11;13(4):e0195659. doi: 10.1371/journal.pone.0195659 (PMC5895053; doi:10.1371/journal.pone.0195659)
Supplement: S1 Appendix — (DOCX) [file pone.0195659.s002.docx]

**S1 Appendix. Search strategy.**

1. PubMed：

(((((delirium) OR "cognitive dysfunction")) AND (((inflammatory or inflammation)) OR cytokines [MeSH Terms]))) AND postoperative Filters: Humans----154

(((postoperative) AND ((delirium) OR "cognitive dysfunction"))) AND ((Inflammation Mediators [MeSH Terms]) OR cytokines[MeSH Terms]) Filters: Humans----64

1. Embsae：551

'cognitive defect'/exp OR 'cognitive defect' OR 'delirium'/exp OR delirium AND postoperati* AND (inflammat* OR 'cytokine'/exp OR cytokine) AND [humans]/lim

1. Cochrane：23

#1= MeSH descriptor: [Inflammation Mediators] explode all trees

#2= MeSH descriptor: [Cytokines] explode all trees

#3= postoperative (Word variations have been searched)

#4= delirium (Word variations have been searched)

#5= cognition (Word variations have been searched)

#6=#3 and #4

#7=#3 and #5

#8=#6 or #7

#9=#1 or #2

#10=#8 and #9

1. Web of Science: 616

#1=TOPIC: (postoperati*)

#2=TOPIC: (cognitive dyfunction) *OR* TORPIC: (delirium)

#3=TOPIC: (inflammat*) *OR* TORPIC: (cytokines)

#4=#1 AND #2

#5=#4 AND #3
