# Supplementary material for: Inflammatory markers in postoperative delirium (POD) and cognitive dysfunction (POCD): A meta-analysis of observational studies
Source: PLoS One. 2018 Apr 11;13(4):e0195659. doi: 10.1371/journal.pone.0195659 (PMC5895053; doi:10.1371/journal.pone.0195659)
Supplement: S1 Table — (DOCX) [file pone.0195659.s003.docx]

**S1 Table. Characteristics of the association studies that were included in the meta-analysis and that examined the peripheral and/or the CSF inflammatory markers.**

| **ID** | **Author /Year** | **Country** | **Design** | **No. of case /control** | **Gender ratio (%male) in case /control** | **Age (years) of cases /controls** | **Anesthesia** | **Surgery** | **Biomarker(s) studied** | **Diagnostic criteria** | **Diagnosis** |
| --- | --- | --- | --- | --- | --- | --- | --- | --- | --- | --- | --- |
| 1 | Baranyi 2012 ^[21]^ | Germany | cohort | 11 /23 | 54.4% /69.6% | 68.5±7.3 /68.0±10.9 | GA | cardiac surgery with CPB | CRP | DRS | POD |
| 2 | Burkhart 2010 ^[22]^ | Switzerland | cohort | 35 /78 | 69% /67.9% | 76.4±5.21 /73.4±5.42 | GA | cardiac surgery with CPB | CRP | CAM | POD |
| 3 | Cape 2014 ^[23]^ | UK /Netherlands | cohort | 19 /24 | 26% /25% | 81.3±6 /81.3±8.6 | RA | hip arthroplasty | IL-1𝛽,  IL-1ra, IGF-1;  (CSF)  IL-1𝛽,  IL-1ra | CAM | POD |
| 4 | Capri 2014 ^[24]^ | Italy | case- control | 37 /37 | 54% /46% | 79.2±6.7 /76.4±6.7 | / | non-cardiac surgery | IL-2, IL-6, IL-8, IL-10 | CAM, DRS | POD |
| 5 | Cerejeira 2013 ^[25]^ | Portugal | cohort | 37 /64 | 40.54% /54.69% | 73.65±5.87 /72.69±6.53 | GA /RA | hip arthroplasty | cortisol, IGF-1, | CAM, DSM-IV-TR | POD |
| 6 | Cerejeira 2012 ^[26]^ | Portugal | cohort | 37 /64 | 40.5% /54.7% | 73.6±5.9 /72.7±6.5 | GA /RA | arthroplasty | CRP,  IL-1𝛽, TNF-𝛼,  IL-6, IL-8, IL-10 | CAM, DSM-IV-TR | POD |
| 7 | Chu 2016 ^[27]^ | China | cohort | 23 /80 | 78.3% /72.5% | 81.13±5.05 /81.91±3.63 | GA /RA | vertebral, knee, or hip surgery | IGF-1 | CAM, DSM-IV-TR | POD |
| 8 | Çinar 2014 ^[28]^ | Turkey | cohort | 15 /20 | 46.7% /95% | 69.0±9.6 /60.7±11.9 | GA | CABG | CRP,  TNF-𝛼, IGF-1 | DRS-R-98, DSM-IV-TR | POD |
| 9 | Guenther 2013 ^[29]^ | Germany | cohort | 69 /146 | 63.8% /67.8% | 73.3 (71.2-75.4) /68.5 (67.0-70.0) | GA | cardiac surgery with CPB | CRP | CAM | POD |
| 10 | Kazmierski 2013 ^[30]^ | Poland | cohort | 41 /72 | 70.3% /84.72% | 68.8 (64-74) /61.5 (58-67.5) | GA | CABG with CPB | cortisol | CAM | POD |
| 11 | Kazmierski 2014 ^[31]^ | Poland | cohort | 41 /72 | 70.3% /84.72% | 68.8 (64-74) /61.5 (58-67.5) | GA | CABG with CPB | TNF-𝛼 | CAM | POD |
| 12 | Lee 2011 ^[32]^ | Korea | cohort | 18 /47 | 44.4% /29.8% | 81.7±6.35 /75.0±7.83 | / | hip surgery | CRP | DSM-IV,  K-DRS-R-98 | POD |
| 13 | Liu 2013 ^[33]^ | China | cohort | 50 /288 | 54% /56.6% | 74±6 /71±7 | GA /RA | non-cardiac surgery | IL-6 | CAM | POD |
| 14 | Neerland 2016 ^[34]^ | Norway /UK | cohort | 71 /78 | 30% /20% | 85(80-89) /83(71-88) | RA | hip surgery | (serum, CSF) CRP,  IL-6 | CAM | POD |
| 15 | Plaschke 2010 ^[35]^ | Germany | case- control | 32 /82 | / | 73.3±6.0 /67.3±9.3 | GA | CABG | CRP | CAM | POD |
| 16 | Rudolph 2008 ^[36]^ | USA | case- control | 12 /12 | 92% /75% | 74.7±7.0 /73.9±8.4 | GA | cardiac surgery | IL-1𝛽,  IL-1ra,  IL-6, IL-8, TNF-𝛼 | CAM | POD |
| 17 | Shen 2016 ^[37]^ | China | cohort | 36 /104 | 47.2% /41.3% | 73.8±5.9 /68.8±7.0 | GA | open abdominal surgery | IGF-1,  IL-6, CRP | DRS-R-98 | POD |
| 18 | van 2010 ^[38]^ | Netherlands | cohort | 62 /58 | 26% /40% | 84.8±6.9 /82.9±7.9 | GA /RA | hip surgery | cortisol,  IL-6, IL-8,  S-100𝛽 | CAM | POD |
| 19 | van 2008 ^[39]^ | Netherlands | cohort | 50 /48 | 26% /37.5% | 84.6±7.1 /83.2±6.7 | GA /RA | hip surgery | IL-6, IL-8 | CAM | POD |
| 20 | Westhoff 2013 ^[40]^ | Netherlands | cohort | 23 /38 | 20.1% /31.6% | 84.6±5.2 /82.9±4.5 | RA | hip surgery | CRP, IL-6; (CSF)  IL-1ra,  IL-6 | CAM | POD |
| 21 | Yen 2016 ^[41]^ | Singapore | cohort | 22 /76 | 41% /50% | 72.5±4.4 /73.7±5.2 | / | knee arthroplasty | IGF-1 | DSM | POD |
| 22 | Ren Q 2015 ^[42]^ | China | cohort | 93 /721 | 34.4% /41.1% | 66.0±15.3 /55.7±14.9 | GA /RA | cardiac and non-cardic surgery | CRP | CAM | POD |
| 23 | Liu WH 2012 ^[43]^ | China | case- control | 42 /42 | 40.5% /42.9% | 49.0±5.7 /44.9±16.0 | GA | non-cardic surgery | S-100𝛽, NSE | CAM | POD |
| 24 | Liu C 2013 ^[44]^ | China | case- control | 16 /50 | 16.7% /53.0% | 63.25±2.58 /50.26±1.95 | GA | endovascular exclusion for aortic dissection | CRP, cortisol | CAM | POD |
| 25 | Zhang TJ 2004 ^[45]^ | China | cohort | 7 /23 | 42.9% /43.5% | / | GA | CABG | S-100𝛽, NSE | CAM | POD |
| 26 | Yang YL 2014 ^[46]^ | China | cohort | 20 /20 | 45% /40% | 80±5 /81±4 | GA | hip replacement | S-100𝛽, NSE | CAM | POD |
| 27 | Lin JH 2016 ^[47]^ | China | case- control | 20 /77 | 45% /40.3% | 79±6 /78±7 | GA /RA | hip surgery | S-100𝛽, NSE, BDNF | CAM | POD |
| 28 | Jiang HB 2014 ^[48]^ | China | case- control | 89 /97 | / | / | RA | hip surgery | CRP | CAM | POD |
| 29 | Chen MM 2016 ^[49]^ | China | cohort | 56 /82 | 42.9% /41.5% | 78.92±3.97 /75.03±5.28 | RA | hip surgery | (CSF) CRP | CAM | POD |
| 30 | Sun 2016 ^[50]^ | China | cohort | 56 /56 | 48.2% /41.1% | 73.2±6.5 /72.7±8.3 | GA | oral cancer patients who underwent tumor resection surgery | CRP, IL-6 | CAM | POD |
| 31 | Goettel 2017 ^[51]^ | Switzerland | cohort | 38 /44 | 66% /57% | 76(68-78) /70(67-76) | GA | non-cardiac surgery | CRP | CERAD-NAB,  Trail Making Tests, Phonemic Fluency Test | POCD |
| 32 | Burkhart 2011 ^[52]^ | Switzerland | cohort | 23 /27 | 61% /63% | 74(70-80) /71(67-74) | GA | non-cardiac surgery | CRP, S100𝛽, NSE | CERAD-NAB | POCD |
| 33 | Li 2013 ^[53]^ | China | cohort | 11 /14 | 90.9% /71.4% | 46.45±8.56 /43.79±9.33 | GA | liver transplantation | CRP | MMSE | POCD |
| 34 | Li 2012 ^[54]^ | China | cohort | 17 /20 | 58.8% /60.0% | >60 />60 | GA | total hip-replacement | S-100𝛽,  IL-6 | Digit Symbol Substitution Test, Concentration Endurance Test, Number Connection Test | POCD |
| 35 | Lin 2014 ^[55]^ | China | cohort | 50 /20 | 64% /75% | 66.1±4.1 /67.2±3.8 | GA | gastrointestinal surgery | IL-6 | HVLT-R, BVMT-R, Trail Making Test, Benton Judgment of Line Orientation, Digit Span Test, Symbol-Digit Modalities Test | POCD |
| 36 | Ramlawi 2006 ^[56]^ | USA | cohort | 16 /24 | 81.3% /91.7% | 69.1±10.5 /66.0±10.6 | GA | cardiac surgery with CPB | CRP,  S-100𝛽,  IL-6, NSE | Trailmaking A & B, HVLT, Digit Span, Boston Naming Test, Semantic Fluency, Phonemic Fluency, Wechsler Test of Adult Reading, Stroop | NCD |
| 37 | Wu 2016 ^[57]^ | China | cohort | 29 /81 | 44.8% /37.0% | 71.8±6.5 /67.4±7.0 | GA | laparoscopic surgery for colon cancer | CRP, IL-6, TNF-𝛼 | CANTAB test | POCD |
| 38 | She YJ 2014 ^[58]^ | China | cohort | 17 /56 | / | 78±5 /73±4 | RA | hip replacement | TNF-𝛼,  IL-1𝛽, IL-6 | MMSE | POCD |
| 49 | Zhang J 2014 ^[59]^ | China | cohort | 48 /60 | 47.9% /45% | 76.5±11.0 /70.3±10.2 | GA | non-cardiac surgery | CRP,  IL-1𝛽, TNF-𝛼,  IL-6 | MMSE | POCD |
| 40 | Zhang Y 2015 ^[60]^ | China | cohort | 19 /58 | 68.4% /62.1% | 73.3±4.8 /69.3±4.6 | GA | colorectal surgery | IL-6, BDNF | MMSE | POCD |
| 41 | Zhang FF 2012 ^[61]^ | China | cohort | 19 /26 | / | 72.3±5.2 /70.4±4.9 | GA | total knee arthroplasty | S-100𝛽,  IL-6, IL-1𝛽,  TNF-𝛼 | MMSE | POCD |
| 42 | Shi LY 2012 ^[62]^ | China | cohort | 17 /23 | 35.3% /65.2% | 61.5±12.3 /53.9±16.0 | GA | orthopedic surgery | IL-6, IL-8, IL-1ra | BCAI | POCD |
| 43 | Yang ZY 2010 ^[63]^ | China | cohort | 18 /21 | / | ≧70 /≧70 | GA | orthopedic surgery | IL-1𝛽, IL-6, TNF-𝛼 | MMSE | POCD |
| 44 | Jia N 2017 ^[64]^ | China | cohort | 19 /70 | 52.6% /65.7% | 68.9±5.6 /68.6±9.2 | GA | orthopedic surgery | IL-1𝛽, IL-6, TNF-𝛼 | MMSE | POCD |
| 45 | Zheng X 2014 ^[65]^ | China | cohort | 17 /46 | 29.4% /26.1% | 14±2 /14±2 | GA | orthopedic surgery | CRP | Semantic Fluency, Phonemic Fluency, Stroop, Digit Span, Trail Making Test, Symbol-Digit Test | POCD |
| 46 | Chen YJ 2011 ^[66]^ | China | cohort | 12 /28 | 41.7% /46.4% | 71.8±4.3 /69.7±4.6 | GA | abdominal surgery | CRP, IL-6 | MMSE | POCD |
| 47 | Ma J 2014 ^[67]^ | China | cohort | 27 /43 | 44.4% /53.5% | 71.2±11.3 /68.5±9.1 | GA | hip arthroplasty or lumbar pedicle screw fixation | IL-1𝛽, IL-6, TNF-𝛼 | MMSE | POCD |
| 48 | Gao ZJ 2014 ^[68]^ | China | case- control | 32 /26 | / | 58.8±5.9 /57.8±7.1 | GA | CABG | IL-6,  S-100𝛽, NSE | Digit Span, Symbol-Digit Test, Trail Making Test, Self- Rating Depression Scale | POCD |
| 49 | Beloosesky 2007 ^[69]^ | Israel | cohort | 12 /29 | / | / | / | hip surgery | CRP,  IL-1𝛽, IL-6, IL-8,  IL-10,  IL-1ra, TNF-𝛼 | MMSE | IMS |
| 50 | Ramlawi 2006 ^[70]^ | Israel | Cohort | 17 /25 | 82.4% /72% | 68.2±10.7 /65.7±10.5 | GA | cardiac surgery | CRP,  IL-1𝛽,  IL-10 | Trailmaking A & B, HVLT, Digit Span, Boston Naming Test, Semantic Fluency, Phonemic Fluency, Weschler Test of Adult Reading, Stroop | NCD |
| 51 | Hu XM 2015 ^[71]^ | China | cohort | 18 /62 | 61.1% /64.5% | 75.62±5.94 /70.28±4.65 | RA | hip replacement | cortisol | MMSE | POCD |
| 52 | Li YC  2011 ^[72]^ | China | cohort | 17 /20 | 58.8% /60.0% | >60 | GA | total  hip-replacement | CRP,  IL-1𝛽, TNF-𝛼,  IL-6 | MMSE | POCD |
| 53 | Zhang 2015 ^[73]^ | China | case- control | 20 /43 | 45% /51.2% | 68±2.54 /58.91±2.03 | GA | lumber discectomy | CRP, IL-6, IL-10 | MoCA | POCD |
| 54 | Zhang Q 2016 ^[74]^ | China | cohort | 30 /120 (POD);  24 /55 (POCD) | 30% /43.3% (POD);  37.5% /49.1% (POCD) | / | GA | non-cardic surgery | IL-6,  TNF-𝛼 | CAM-ICU, MMSE | POD  /POCD |

Year: published year;

Design: types of research design;

No. of case/control: the number of patients with POD/POCD versus without POD/POCD;

Gender ratio (%male) in case/control: the percentage of male patients with POD/POCD versus without POD/POCD;

Age (years) of Cases / Controls: the age of patients with POD/POCD versus without POD/POCD in year;

Anesthesia: type of anesthesia;

GA: general anesthesia;

RA: regional anesthesia;

Surgery: type of surgery;

CPB: cardiopulmonary bypass;

CABG: coronary artery bypass grafting;

CSF: cerebrospinal fluid;

CRP: C-reactive protein;

IL-1β: interleukin-1β;

IL-1ra: interleukin-1 receptor antagonist;

IL-6: interleukin-6;

IL-8: interleukin-8;

IL-10: interleukin-10;

IGF-1: Insulin-like growth factor-1;

NSE: neuron specific enolase;

S-100β: S-100β protein;

TNF-α: tumor necrosis factor-α;

CAM: Confusion Assessment Method

CAM-ICU: The Confusion Assessment Method for the Intensive Care Unit

MMSE: Mini-mental State Examination

DSM: Diagnostic and Statistical Manual of Mental Disorders

DSM-IV-TR: Diagnostic and Statistical Manual of Mental Disorders (Fourth Edition, Text Revision)

DRS: Delirium Rating Scale

DRS-R-98: Delirium Rating Scale-Revised-98

K-DRS-98: Korean version of the Delirium Rating Scale-Revised-98

CERAD-NAB: Consortium to Establish a Registry for Alzheimer’s Disease-Neuropsychological Assessment Battery

HVLT-R: Hopkin Verbal Learning Test-Revised

BVMT-R: Brief Visuospatial Memory Test-Revised

CANTAB test: the Cambridge Neuropsychological Test Automated Battery

MoCA: Montreal Cognitive Assessment

BCAI: Battery of Cognitive Assessment Instruments for Elderly

POD: post-operative delirium

POCD: post-operative cognitive dysfunction

IMS: impaired mental status

NCD: neurocognitive deficiency
