# Supplementary material for: Inflammatory markers in postoperative delirium (POD) and cognitive dysfunction (POCD): A meta-analysis of observational studies
Source: PLoS One. 2018 Apr 11;13(4):e0195659. doi: 10.1371/journal.pone.0195659 (PMC5895053; doi:10.1371/journal.pone.0195659)
Supplement: S2 Table — (DOCX) [file pone.0195659.s004.docx]

**S2A Table. Newcastle-Ottawa quality assessment scale for evaluation to the quality of case control studies**

|  |  | **Selection** | | | | **Comparability** | | **Exposure** | | |  |
| --- | --- | --- | --- | --- | --- | --- | --- | --- | --- | --- | --- |
| ID | First author | #1 | #2 | #3 | #4 | #5 | #6 | #7 | #8 | #9 | Total |
| 1 | Capri 2014 | ★ |  |  | ★ |  |  | ★ | ★ | ★ | 5 |
| 2 | Plaschke 2010 | ★ | ★ | ★ |  |  |  | ★ | ★ | ★ | 6 |
| 3 | Rudolph 2008 | ★ |  |  | ★ |  |  | ★ | ★ | ★ | 5 |
| 4 | Zhang 2015 | ★ |  | ★ | ★ | ★ |  | ★ | ★ |  | 6 |
| 5 | Liu WH 2012 | ★ |  |  | ★ |  |  | ★ | ★ |  | 4 |
| 6 | Liu C 2013 | ★ |  |  | ★ | ★ |  | ★ | ★ |  | 5 |
| 7 | Lin JH 2016 | ★ |  |  | ★ |  |  | ★ | ★ |  | 4 |
| 8 | Lin GX 2014 | ★ |  |  | ★ |  | ★ | ★ | ★ |  | 5 |
| 9 | Jiang HB 2014 | ★ |  |  |  |  |  | ★ | ★ |  | 3 |
| 10 | Gao ZJ 2014 | ★ |  | ★ | ★ | ★ | ★ | ★ | ★ |  | 7 |

#1. Is POD/POCD definition adequate?

#2. Representativeness of patients with POD/POCD

#3. Selection of the Comparative patients without POD/POCD

#4. Definition of Controls

#5. Study controls for age

#6. Study controls for Gender/Type of surgery/Type of anesthesia

#7. Ascertainment of POD/POCD station

#8. Same method of ascertainment for POD/POCD station

#9. Same non-response rate tor cases and controls

**S2B Table. Newcastle-Ottawa quality assessment scale for evaluation to the quality of cohort studies**

|  |  | **Selection** | | | | **Comparability** | | **Outcome** | | |  |
| --- | --- | --- | --- | --- | --- | --- | --- | --- | --- | --- | --- |
| ID | First author | #1 | #2 | #3 | #4 | #5 | #6 | #7 | #8 | #9 | Total |
| 1 | Baranyi 2012 | ★ | ★ | ★ | ★ |  |  | ★ | ★ | ★ | 7 |
| 2 | Burkhart 2010 | ★ | ★ | ★ | ★ |  | ★ | ★ | ★ | ★ | 8 |
| 3 | Burkhart 2011 | ★ | ★ | ★ |  |  |  |  | ★ | ★ | 5 |
| 4 | Cape 2014 | ★ | ★ | ★ |  |  |  |  | ★ | ★ | 5 |
| 5 | Cerejeira 2013 | ★ | ★ | ★ | ★ |  |  |  | ★ |  | 5 |
| 6 | Cerejeira 2012 | ★ | ★ | ★ | ★ |  |  |  | ★ | ★ | 6 |
| 7 | Chu 2016 | ★ | ★ | ★ | ★ |  |  |  | ★ |  | 5 |
| 8 | Çinar 2014 | ★ | ★ | ★ |  |  |  |  | ★ | ★ | 5 |
| 9 | Goettel 2017 | ★ | ★ | ★ | ★ | ★ |  | ★ | ★ | ★ | 8 |
| 10 | Guenther 2013 | ★ | ★ | ★ |  | ★ | ★ |  | ★ | ★ | 7 |
| 11 | Kazmierski 2013 | ★ | ★ | ★ | ★ |  | ★ | ★ | ★ | ★ | 8 |
| 12 | Kazmierski 2014 | ★ | ★ | ★ | ★ | ★ | ★ | ★ | ★ |  | 8 |
| 13 | Lee 2011 | ★ | ★ | ★ | ★ |  |  |  | ★ |  | 5 |
| 14 | Li 2013 | ★ | ★ | ★ | ★ |  |  | ★ | ★ | ★ | 7 |
| 15 | Li 2012 | ★ | ★ | ★ | ★ |  |  | ★ | ★ | ★ | 7 |
| 16 | Lin 2014 | ★ | ★ | ★ | ★ | ★ |  | ★ | ★ |  | 7 |
| 17 | Liu 2013 | ★ | ★ | ★ |  |  |  | ★ | ★ | ★ | 6 |
| 18 | Neerland 2016 | ★ | ★ | ★ |  |  |  | ★ | ★ |  | 5 |
| 19 | Ramlawi 2006 | ★ | ★ | ★ | ★ |  |  | ★ | ★ | ★ | 7 |
| 20 | Shen 2016 | ★ | ★ | ★ | ★ | ★ |  |  | ★ | ★ | 7 |
| 21 | Sun 2016 | ★ | ★ | ★ | ★ |  |  | ★ | ★ | ★ | 7 |
| 22 | van 2010 | ★ | ★ | ★ |  |  |  | ★ | ★ | ★ | 6 |
| 23 | van 2008 | ★ | ★ | ★ |  |  |  | ★ | ★ | ★ | 6 |
| 24 | Westhoff 2013 | ★ | ★ | ★ | ★ |  |  | ★ | ★ | ★ | 7 |
| 25 | Wu 2016 | ★ | ★ | ★ | ★ | ★ |  | ★ | ★ |  | 7 |
| 26 | Yen 2016 | ★ | ★ | ★ | ★ |  |  | ★ | ★ |  | 6 |
| 27 | Ren Q 2015 | ★ | ★ | ★ |  | ★ | ★ | ★ | ★ | ★ | 8 |
| 28 | She YJ2014 | ★ | ★ | ★ | ★ |  |  |  | ★ |  | 5 |
| 29 | Zhang J 2014 | ★ | ★ | ★ | ★ | ★ |  |  |  |  | 5 |
| 30 | Zhang Y 2015 | ★ | ★ | ★ | ★ |  |  |  | ★ |  | 5 |
| 31 | Zhang TJ 2004 | ★ | ★ | ★ |  |  |  |  | ★ | ★ | 5 |
| 32 | Zhang FF 2012 | ★ | ★ | ★ | ★ |  |  | ★ | ★ |  | 6 |
| 33 | Shi LY 2012 | ★ | ★ | ★ | ★ |  |  | ★ | ★ | ★ | 7 |
| 34 | Yang YL 2014 | ★ | ★ | ★ |  |  |  | ★ | ★ |  | 5 |
| 35 | Yang ZY 2010 | ★ | ★ | ★ |  |  |  | ★ | ★ |  | 5 |
| 36 | Jia N 2017 | ★ | ★ | ★ | ★ |  |  | ★ | ★ | ★ | 7 |
| 37 | Zheng X 2014 | ★ | ★ | ★ | ★ |  |  | ★ | ★ | ★ | 7 |
| 38 | Chen YJ 2011 | ★ | ★ | ★ | ★ |  |  |  | ★ | ★ | 6 |
| 39 | Zhang Q 2016 | ★ | ★ | ★ | ★ | ★ |  | ★ | ★ |  | 7 |
| 40 | Ma J 2014 | ★ | ★ | ★ | ★ |  |  | ★ | ★ | ★ | 7 |
| 41 | Beloosesky 2007 | ★ | ★ | ★ |  |  |  | ★ | ★ | ★ | 6 |
| 42 | Li YC  2011 | ★ | ★ | ★ | ★ |  |  |  | ★ |  | 5 |
| 43 | Basel 2006 | ★ | ★ | ★ | ★ |  |  | ★ | ★ | ★ | 7 |
| 44 | Chen MM 2016 | ★ | ★ | ★ |  |  |  | ★ | ★ | ★ | 6 |

#1. Representativeness of patients with POD/POCD

#2. Selection of the Comparative patients without POD/POCD

#3. Ascertainment of POD/POCD station

#4. Demonstration that POD/POCD was not present at start of study

#5. Study controls for age

#6. Study controls for Gender/Type of surgery/Type of anesthesia

#7. Independent or blind assessment stated in the paper, or confirmation of POD/POCD by reference to secure records

#8. Was follow-up long enough for POD/POCD to occur

#9. Adequacy of follow up of POD/POCD
