# Supplementary material for: Inflammatory markers in postoperative delirium (POD) and cognitive dysfunction (POCD): A meta-analysis of observational studies
Source: PLoS One. 2018 Apr 11;13(4):e0195659. doi: 10.1371/journal.pone.0195659 (PMC5895053; doi:10.1371/journal.pone.0195659)
Supplement: S3 Table — (DOCX) [file pone.0195659.s005.docx]

**S3 Table. Subgroup analyses for the assessment of impact of surgery type, anesthesia, and ethnicity on peripheral CRP and IL-6 levels perioperatively.**

| **Subgroups** | **Studies with** | **No. of studies** | **Heterogeneity** | | **Std. Mean Difference [95% CI] between case and control subjects** |
| --- | --- | --- | --- | --- | --- |
|  |  |  | ***P*** | **I^2^** |  |
| CRP levels before surgery in POD /non-POD | | | | | |
| Surgery | Cardiac surgery | 3 | 0.000 | 95.6% | 0.292 [-1.253, 1.837]; *P*=0.711; REM |
|  | Non-cardiac surgery | 6 | 0.000 | 95.1% | 1.192 [0.210, 7.188]; *P*=0.017; REM |
| Ethnicity | Caucasians | 6 | 0.000 | 93.0% | 0.196[-0.579, 0.971]; *P*=0.620; REM |
|  | Chinese | 3 | 0.000 | 97.0% | 2.470[0.395, 4.544]; *P*=0.020; REM |
| Anesthesia | General anesthesia | 5 | 0.000 | 96.4% | 1.461[0.177,2.745]; *P*=0.026; REM |
|  | Regional anesthesia | 2 | 0.976 | 0.0% | 0.208[-0.182,0.599]; *P*=0.296; REM |
|  | Mixed anesthesia | 1 | / | / | / |
| Overall | | 9 | 0.000 | 94.8% | 0.883[0.130, 1.637]; *P*=0.022; REM |
| CRP levels before surgery in POCD /non-POCD | | | | | |
| Surgery | Cardiac surgery | 2 | 0.841 | 0.0% | -1.414[-1.908, -0.920]; *P*=0.000; REM |
|  | Non-cardiac surgery | 8 | 0.386 | 5.7% | 0.142[-0.051, 0.334]; *P*=0.149; REM |
| Ethnicity | Caucasians | 4 | 0.000 | 89.6% | -0.504 [-1.521, 0.512]; *P*=0.331; REM |
|  | Chinese | 6 | 0.312 | 15.8% | 0.084 [-0.144, 0.311]; *P*=0.470; REM |
| Overall | | 10 | 0.000 | 77.9% | -0.133[-0.512, 0.246]; *P*=0.492; REM |
| IL-6 levels before surgery in POD /non-POD | | | | | |
| Surgery | Cardiac surgery | 1 | / | / | / |
|  | Non-cardiac surgery | 6 | 0.006 | 69.2% | 0.481 [0.172, 0.791]; *P*=0.002; REM |
| Ethnicity | Caucasians | 5 | 0.006 | 72.6% | 0.277 [-0.166, 0.720]; *P*=0.220; REM |
|  | Chinese | 2 | 0.016 | 82.8% | 0.594 [0.054, 0.717]; *P*=0.051; REM |
| Anesthesia | General anesthesia | 2 | 0.001 | 90.7% | 0.194 [-1.300,1.687]; *P*=0.799; REM |
|  | Regional anesthesia | 2 | 0.076 | 68.2% | 0.670 [0.028,1.311]; *P*=0.041; REM |
|  | Mixed anesthesia | 2 | 0.170 | 46.9% | 0.153 [-0.189,0.495]; *P*=0.381; REM |
| Overall | | 7 | 0.001 | 73.2% | 0.386 [0.054, 0.717]; *P*=0.022; REM |
| IL-6 levels before surgery in POCD /non-POCD | | | | | |
| Surgery | Cardiac surgery | 2 | 0.244 | 26.3% | 0.107 [-0.434, 0.647]; *P*=0.699; REM |
|  | Non-cardiac surgery | 14 | 0.000 | 64.7% | 0.087 [-0.158, 0.332]; *P*=0.485; REM |
| Ethnicity | Caucasians | 2 | 0.244 | 26.3% | 0.107 [-0.434, 0.647]; *P*=0.699; REM |
|  | Chinese | 14 | 0.000 | 64.7% | 0.087 [-0.158, 0.332]; *P*=0.485; REM |
| Overall | | 16 | 0.001 | 60.7% | 0.089 [-0.133, 0.311]; *P*=0.433; REM |
